# *Bifidobacterium* catabolism of human milk oligosaccharides overrides endogenous competitive exclusion driving colonization and protection

**DOI:** 10.1080/19490976.2021.1986666

**Published:** 2021-10-27

**Authors:** Britta E. Heiss, Amy M. Ehrlich, Maria X. Maldonado-Gomez, Diana H. Taft, Jules A. Larke, Michael L. Goodson, Carolyn M. Slupsky, Daniel J. Tancredi, Helen E. Raybould, David A. Mills

**Affiliations:** a Department of Food Science and Technology, University of California-Davis, Davis, CA, USA; b Foods for Health Institute, University of California-Davis, Davis, CA, USA; c Department of Anatomy, Physiology, and Cell Biology, School of Veterinary Medicine, University of California-Davis, Davis, CA, USA; d Department of Nutrition, University of California-Davis, Davis, CA, USA; e Center for Healthcare Policy and Research, Department of Pediatrics, University of California-Davis, Sacramento, CA, USA

**Keywords:** Gut microbiota, human milk oligosaccharides, *Bifidobacterium*, colonization, probiotics

## Abstract

Understanding how exogenous microbes stably colonize the animal gut is essential to reveal mechanisms of action and tailor effective probiotic treatments. *Bifidobacterium* species are naturally enriched in the gastrointestinal tract of breast-fed infants. Human milk oligosaccharides (HMOs) are associated with this enrichment. However, direct mechanistic proof of the importance of HMOs in this colonization is lacking given milk contains additional factors that impact the gut microbiota. This study examined mice supplemented with the HMO 2ʹfucosyllactose (2ʹFL) together with a 2ʹFL-consuming strain, *Bifidobacterium pseudocatenulatum* MP80. 2ʹFL supplementation creates a niche for high levels of *B.p*. MP80 persistence, similar to *Bifidobacterium* levels seen in breast-fed infants. This synergism impacted gut microbiota composition, activated anti-inflammatory pathways and protected against chemically-induced colitis. These results demonstrate that bacterial-milk glycan interactions alone drive enrichment of beneficial *Bifidobacterium* and provide a model for tunable colonization thus facilitating insight into mechanisms of health promotion by bifidobacteriain neonates.

## Introduction

Probiotics are commercially available supplements that are increasingly examined for their role in preventing a number of diseases including necrotizing enterocolitis and antibiotic-associated diarrhea among others.^1,2^ Despite potential benefits, the specific role of probiotics in mitigating diseases remains controversial.^3^ One aspect of this debate is the relative lack of persistence of supplemented probiotics in gut ecosystems due to the inherent heterogeneity and colonization resistance of the human gut microbiota.^4^ Most probiotic species survive passage through the intestinal tract, but persistence of the microbe, that is, detection of elevated levels of the strain post-bacterial supplementation due to substantive growth and metabolism, is infrequent.^5^ The persistence of a species is defined as the time between its emergence and extinction within a defined region.^6^ Persistence time of a species is determined by the environmental conditions in the habitat, presence of additional species, and access to nutrient resources. In the case of bacterial persistence, specific conditions permit the microbe to replicate at an equal or greater rate than washout.^5^ In short, the low abundance, lack of persistence, and likely low metabolite production from a probiotic population that diminishes after supplementation ceases are potential factors in the variable impact of probiotics on health outcomes. Thus, one means to address these concerns is to identify a mechanism that results in multiplication and persistence of specific microbes in the gastrointestinal tract, thereby allowing more robust examination of host-probiotic interactions and facilitating mechanistic exploration of the resulting health outcomes. A recent study demonstrated in mice that provision of a unique dietary carbohydrate, also known as a privileged nutrient niche, can facilitate engraftment of *Bacteroides* strains competent in catabolism of such carbohydrates.^7,8^ However, no examination of colonization-associated health outcomes, a key factor in defining probiotics, was undertaken.

One model for the sustained diet-driven persistence of a specific beneficial bacterial taxa in the human gut is the common observation of *Bifidobacterium* enrichment during nursing. Numerous studies have identified associations between early, and predominant, colonization of infant-borne *Bifidobacterium* and beneficial health outcomes in breast-fed infants.^9–14^ A number of studies have illustrated possible mechanisms by which probiotic *Bifidobacterium* impact host health including production of acetate, indole-3-lactic acid, exopolysaccharide, and pili.^15–19^ While *Bifidobacterium* colonization of infants has been associated with positive health outcomes, results on the clinical use of specific *Bifidobacterium* probiotics to address human disease remains varied.^20,21^

While the underlying mechanisms for seeding, expansion, and predominance of *Bifidobacterium* in the infant gut are not fully resolved, human milk oligosaccharides (HMOs) are considered a privileged nutrient enabling enrichment of a HMO-catabolizing *Bifidobacterium* population.^22–24^ HMOs are structurally complex molecules composed of a range of monomers and linkages which require a complex assembly of bacterial glycosyl hydrolases and transport systems to catabolize them, making them a privileged nutrient that few microbes are capable of consuming. Several studies identified associations in breast-fed infants between HMO consumption, enrichment of certain *Bifidobacterium* strains, and higher fecal acetate and lactate (end products of *Bifidobacterium* fermentation).^23,25^ However, considering the constellation of bioactive factors in human milk, notably antimicrobial factors such as lysozyme, lactoferrin and antimicrobial peptides, the magnitude of the effect that HMOs have in the assembly of the developing infant gut microbiome remains unclear.

The aim of this study was to address two associated questions: (1) do HMOs alone act as a privileged nutrient enabling enrichment of a cognate HMO-consuming *Bifidobacterium* in a complex established gut ecosystem of the adult mouse and (2) if *Bifidobacterium* enrichment occurs, does it provide a health benefit? This research is important in establishing the dominant role of HMOs in the colonization of the breast-fed infant gut by *Bifidobacterium*. Moreover, this work illustrates a path whereby provision of a specific nutrient for a supplemented probiotic could drive high-level persistence in the gastrointestinal tract and impact host health.

## Results

### B. pseudocatenulatum persistence is associated with genetic capability to catabolize 2ʹFL

**Figure 1.**
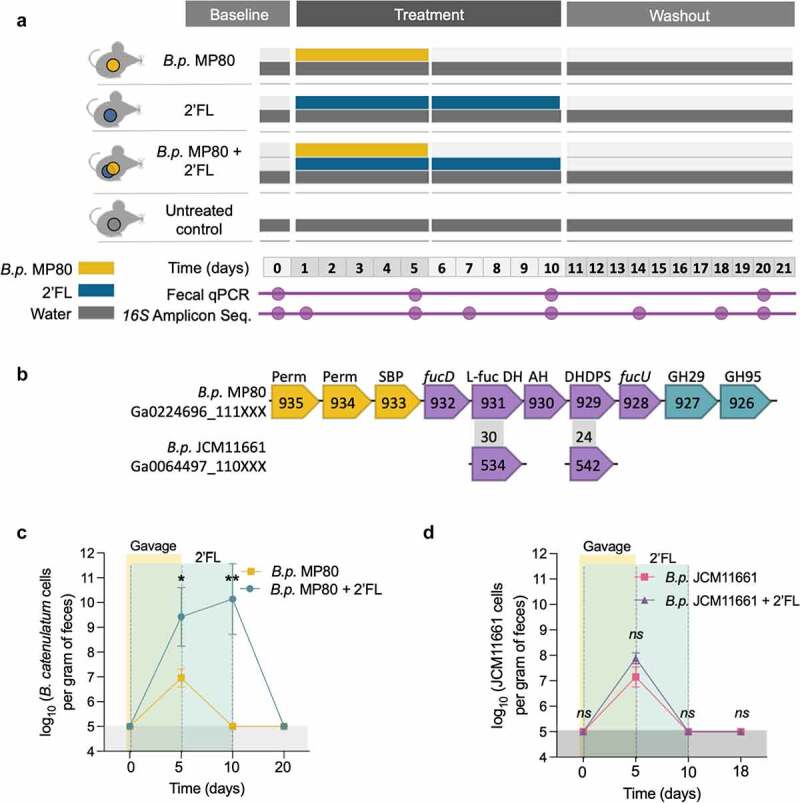
*Bifidobacterium* persistence during 2ʹFL supplementation in mice. (a) Mouse experimental design; (b) fucosylated HMO utilization gene cluster comparison; (c) quantification of *B.p*. MP80 by qPCR in fecal DNA of treatment groups *B.p*. MP80 + 2ʹFL (*n* = 6) and *B.p*. MP80 (*n* = 6); and (d) quantification of *B.p*. JCM11661 by qPCR in fecal DNA of treatment groups *B.p*. JCM11661 + 2ʹFL (*n* = 3) and *B.p*. JCM11661 (*n* = 3). In (a) treatments consisted of 4 groups of mice; untreated = oral gavage of PBS (day 1–5) and drinking water (day 1–20); *B.p*. MP80 = oral gavage of *B.p*. MP80 (day 1–5) and drinking water (day 1–20); 2ʹFL = oral gavage of PBS (day 1–5) and 2ʹFL in drinking water (day 1–10); *B.p*. MP80 + 2ʹFL = oral gavage of *B.p*. MP80 (day 1–5) and 2ʹFL in drinking water (day 1–10); *n* = 6 per treatment group. In (b) arrows represent genes and inset numbers indicate the locus tag number for the respective genome from the IMG/MER databbase hosted by the Joint Genome Institute. Number in gray box indicates percent identity between homologs relative to strain *B.p*. MP80. Colors are indicative of the primary function: oligosaccharide transport (yellow), carbohydrate feeder pathways (purple) and glycosyl hydrolases (blue). Perm: ABC Permease; SBP: Solute Binding Protein; L-Fuc DH: L-fuconate dehydrogenase; DHDPS: Dihydropicolinate synthase; FucU: L-fucose mutarotase. In (c) and (d) day 0: baseline, after acclimation to the animal facility; day 5: final *Bifidobacterium* or PBS gavage day; day 10: final 2ʹFL supplementation day; day 20: last day of 2'FL washout, day before necropsy. One-way ANOVA with multiple comparison testing between treatments at individual time points; * *p* < .05, ** *p* < .01, *** *p* < .001, *ns = *not significant

To determine if the interaction between HMOs and *Bifidobacterium* would enable persistence of the strain, we administered 2ʹ-fucosyllactose (2ʹFL), a predominant HMO in breast milk, and infant-isolate *B. pseudocatenulatum* MP80 to mice (Figure 1a). *B.p*. MP80 grows robustly on 2ʹFL and possesses a unique genetic operon linked to catabolism of this HMO (Figure 1b).^26^ C57BL/6 mice received *B.p*. MP80 for 5 days by oral gavage and simultaneously 2ʹFL was provided in the drinking water (10% w/v; average consumption of 550 mg/day; Figure 1a). 2ʹFL supplementation continued for 5 additional days after *B.p*. MP80 gavage ended. On day 5 (the last day of bacterial oral gavage), *B.p*. MP80 was detected at a high level (>10e10 cells/gram of feces) in mice treated with 2ʹFL, compared to mice receiving *B.p*. MP80 and water (day 5, <10e7 cells/gram of feces, one-way ANOVA, *p* = .008; Figure 1c). After discontinuation of bacterial oral gavage, *B.p* MP80 persisted in mice that continued to receive 2ʹFL, but not in mice that received drinking water alone (day 10, one-way ANOVA, *p* = .0007; Figure 1c). Ten days after the end of 2ʹFL supplementation, *B.p*. MP80 levels were below the limit of detection (day 20, washout; Figure 1c).

We evaluated 2ʹFL catabolism by metabolite profiling of mouse colon contents using ^1^H-NMR spectroscopy. Comparison across treatments resulted in no *p* values <.05 after FDR correction (ANOVA with post hoc Games Howell test; Supplemental Table S2). Due to the small sample size and high number of metabolite features, effect size (Hedge’s g) was calculated to complement hypothesis testing by providing an estimate of treatment effects.^27^ Hedge’s g effect sizes revealed several medium (|g| > .5) and large (|g| > .8) treatment effects (Supplemental Figure S1a-c). Notably, during 2ʹFL supplementation the fucose catabolism end product 1,2-propanediol was elevated in the colon luminal contents of *B.p*. MP80 + 2ʹFL treated mice relative to 2ʹFL alone (Hedge’s g, |g| = 1.48; Supplemental Figure S1d) or untreated control mice (Hedge’s g, |g| = 1.47).

Despite repeated attempts we were unable to transform *B.p*. MP80 for creation of genetic knockouts. Thus, to evaluate whether a 2ʹFL gene cluster is necessary for *Bifidobacterium* persistence, we administered *B. pseudocatenulatum* JCM11661 which lacks α-fucosidases and fails to grow on 2ʹFL, to mice (Figure 1d).^26^ Genome comparison^28^ of *B.p*. JCM11661 to *B.p*. MP80 revealed that 72% of their genomes had a similarity of >75%. *B.p*. JCM11661 was detected during bacterial oral gavage (day 5, >10e8 cells/gram of feces; Figure 1d), but failed to persist during 2ʹFL supplementation alone (day 10; Figure 1d). In addition, there was no significant difference in *B.p*. JCM11611 levels between mice given 2ʹFL or drinking water.

Additional mouse experiments evaluated the frequency of bacterial oral gavage and the concentration of 2ʹFL required for *B.p*. MP80 persistence. Three days of *B.p*. MP80 gavage is sufficient for elevated persistence after bacterial oral gavage discontinuation (day 10, >10e9 cells/g feces) and 10% 2ʹFL yielded the highest persistence (day 10, >10e10 cells/g feces; Supplemental Figure S2a). 2ʹFL at 5% and 2.5% resulted in lower persistence than 10% (day 10, <10e8 cells/g feces), indicating that adjusting 2ʹFL concentration controls strain abundance (Supplemental Figure S2b).

### 2ʹFL driven B.p. MP80 persistence impacts microbial community membership

**Figure 2.**
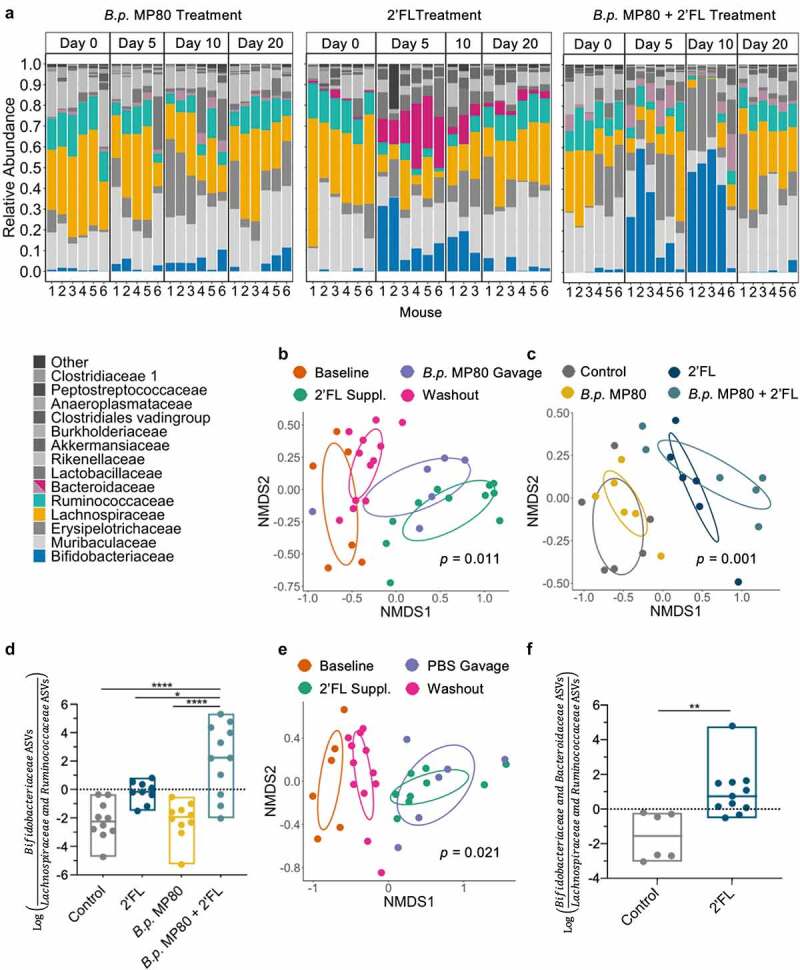
Microbial community structure changes during treatments. (a) Barplots of bacterial family relative abundance for individual mice from treatment groups *B.p*. MP80, 2ʹFL, and *B.p*. MP80 + 2ʹFL (*n* = 6 per treatment) at four time points (day 0: baseline; day 5: final day of *B.p*. MP80 or PBS gavage; day 10: final day of 2ʹFL supplementation; day 20: after 10 days washout of 2ʹFL, day before necropsy). Numbers along the x-axis indicate individual mice. To highlight key bacterial families identified in differential abundance testing, *Bacteroidaceae* is colored deep pink for 2ʹFL treatment, while *Bacteroidaceae* is gray-pink for *B.p*. MP80 and *B.p*. MP80 + 2ʹFL. (b) Non-metric Multi-dimensional Scaling (NMDS) plot of the β-diversity index Bray-Curtis for *B.p*. MP80 + 2ʹFL treatment group, time periods separated by color; (c) NMDS plot of β-diversity index Bray-Curtis on final day of 2ʹFL supplementation (day 10), colored by treatment; (d) log ratio of *Bifidobacteriaceae* relative to low ranked *Lachnospiraceae* and *Ruminococcaceae* ASVs on the final day of 2ʹFL supplementation (day 10); (e) NMDS plot of the β-diversity index Bray-Curtis for 2ʹFL treatment group, time periods separated by color; and (f) 2ʹFL treatment log ratio of *Bifidobacteriaceae* and *Bacteroidaceae* relative to low ranked *Lachnospiraceae* and *Ruminococcaceae* ASVs on the final day of 2ʹFL supplementation (day 10). In (d) and (f) one-way ANOVA with Tukey’s multiple comparisons test; * *p* < .05, ** *p* < .01, *** *p* < .001

To understand the overall impact of persistence of *B.p*. MP80 on α-diversity, microbial community membership, and *Bifidobacterium* levels, *16S rRNA* gene amplicon sequencing was performed. *Bifidobacteriaceae* was elevated from approximately <1% to 40% relative abundance in mice treated with *B.p*. MP80 + 2ʹFL from day 0 (baseline) through day 10 (end of 2ʹFL supplementation) (Figure 2a). In mice treated with *B.p*. MP80 or 2ʹFL alone, relative abundance of *Bifidobacteriaceae* only reached approximately 5% or 11%, respectively (Figure 2a).

A linear regression with sandwich variance estimates to account for heteroskedasticity was used to estimate the effect of the interaction between *B.p*. MP80 in the presence of 2ʹFL on α-diversity in comparison to *B.p*. JCM11611. Administration of *B.p*. MP80 and 2ʹFL is associated with reduced α-diversity (Shannon Index; t-statistic = −3.17, *p* = .013; Supplemental Table S3) compared to *B.p*. JCM11661 and 2ʹFL.

Microbial community structure differed for *B.p*. MP80 + 2ʹFL mice by day as measured by β-diversity (Bray Curtis, PERMANOVA, *p* = .011; Figure 2b). Community structure clearly shifts from baseline (day 0) during days of 2ʹFL supplementation and returns to baseline during washout (Figure 2b). Post-hoc testing for community structure differences between days of 2ʹFL-dependent *B.p*. MP80 persistence (day 10) was significant when compared to baseline (day 0, pairwise PERMANOVA, *p* = .028) and washout (day 20, pairwise PERMANOVA, *p* = .005; Figure 2b). As expected, provision of *B.p*. MP80 to mice generated changes in microbial community structure for treatments *B.p*. MP80 + 2ʹFL and *B.p*. MP80 (Figure 2b and Supplemental Figure S3a). However, a more dramatic disruption in community membership occurred when 2ʹFL was paired with *B.p*. MP80, based on dissimilarity community measures (Bray Curtis, baseline (day 0) vs final *B.p*. MP80 gavage (day 5), Mann-Whitney test, *p* = .022; Supplemental Figure S3b). During *B.p*. MP80 persistence (day 10), comparison of overall microbial community structure by treatment was significant (PERMANOVA, *p* = .001; Figure 2c) although 2ʹFL alone and *B.p*. MP80 + 2ʹFL were not distinct from each other (pairwise PERMANOVA, *p* = .358; Supplemental Table S4).

### B.p. MP80 + 2ʹFL treatment enriches Bifidobacteriaceae relative to Lachnospiraceae and Ruminococcaceae

Microbial differential abundance testing evaluated whether specific ASVs were being increased relative to other taxa. The log ratio of *Bifidobacteriaceae* ASVs to the combination of *Lachnospiraceae* and *Ruminococcaceae* ASVs was identified by Songbird and was significantly increased by treatment during 2ʹFL supplementation (one-way ANOVA, *p* < .001; Figure 2d).^29^ During persistence of *B.p*. MP80, the *Bifidobacteriaceae:Lachnospiraceae* and *Ruminococcaceae* log ratio from *B.p*. MP80 + 2ʹFL treated mice was significantly higher than that found in untreated (Tukey’s test, *p* < .001) and 2ʹFL treated mice (Tukey’s test, *p* = .017; Supplemental Table S5). Absolute abundance measured by qPCR confirms a ~2 log increase of the genus *Bifidobacterium* in *B.p*. MP80 mice during 2ʹFL supplementation (baseline vs day 10, Kruskal Wallis, *p* = .011; Supplemental Figure S3c).

### 2ʹFL enriches Bacteroidaceae and Bifidobacteriaceae relative to Lachnospiraceae and Ruminococcaceae

Although the mouse-associated microbiota has not been naturally selected to catabolize HMOs, we examined the 2ʹFL control group to assess how 2ʹFL may effect change in an established microbiota. β-diversity (Bray Curtis) varied significantly by day (PERMANOVA, *p* = .021; Figure 2e) and 2ʹFL supplemented days were distinct from non-2ʹFL days (pairwise PERMANOVA, *p* = .037). Although no *Bifidobacterium* was provided, increased *Bifidobacteriaceae* relative abundance is noted (Figure 2a). Absolute abundance of the genus *Bifidobacterium* increased by 1–2 logs between baseline (day 0) and day 10 of 2ʹFL supplementation (Kruskal Wallis, *p* = .024; Supplemental Figure S3c). In contrast to untreated control mice, 2ʹFL treatment results in high log ratios of *Bacteroidaceae* and *Bifidobacteriaceae* relative to *Lachnospiraceae* and *Ruminococcaceae* ASVs (student’s t test, *p* = .003; Figure 2f).

### Effects of B.p. MP80 + 2ʹFL treatment in healthy mice

**Figure 3.**
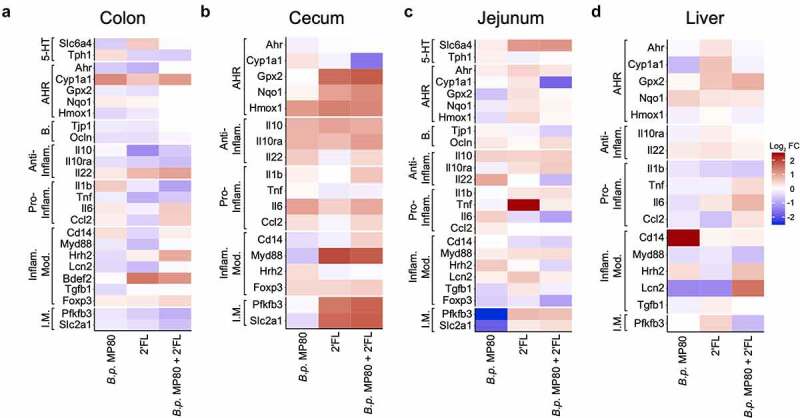
Heat maps of gene expression changes in mice in the (a) distal colon, (b) cecum, (c) jejunum, and (d) liver. Log_2_ transformed fold changes are expressed relative to untreated control mice (*n* = 6 per treatment). Genes were grouped according to known function: serotonin (5-HT) regulatory targets, aryl hydrocarbon receptor (AhR) pathway targets, intestinal barrier markers, anti-inflammatory, pro-inflammatory, inflammatory modulating, and inflammatory metabolism. Statistical analysis for this data is in Supplemental Figures S4-S6 and Supplemental Tables S10-S16

Treatment with *B.p*. MP80, 2ʹFL, or *B.p*. MP80 + 2ʹFL compared to untreated control mice had no significant effect on food or fluid intake, body weight gain, or spleen or liver weights (Supplemental Table S9). Total cecum weight (cecal tissue plus content) was significantly increased in *B.p*. MP80 + 2ʹFL compared to other groups, suggesting fermentation was increased (untreated, *B.p*. MP80- and 2ʹFL-treated mice; *p* < .0001, *p* < .0001, *p* = .0005; Supplemental Table S9). Treatment with *B.p*. MP80 + 2ʹFL induced changes in expression in a number of genes, which varied depending on the region of the gut (Figure 3, Supplemental Tables S10-S16). Changes in gene expression were more evident in the cecum with an increase in expression of both anti-inflammatory and pro-inflammatory markers including MyD88, Nrf2 targets (Gpx2, Hmox1, and Nqo1), and Pfkfb3 and Slc2a1 (Supplemental Figure S4). Treatments had little effect on colon and liver pro- or anti-inflammatory gene expression. *B.p*. MP80 + 2ʹFL reduced expression of Il1b in the colon (*p* = .009; Supplemental Figure S5, Supplemental Tables S10-S11).

### B.p. MP80 + 2ʹFL treatment attenuates DSS-induced colitis

**Figure 4.**
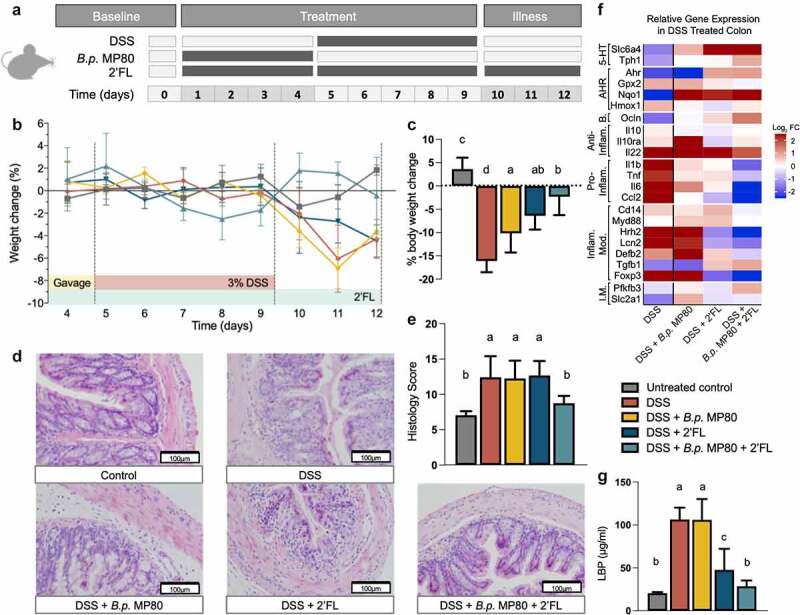
Effects of *B.p*. MP80 and 2ʹFL supplementation in mice challenged with DSS-induced colitis. (a) Experimental timeline (*n* = 6 per treatment); (b) percent change in weight, as compared to the previous day, during and following DSS challenge; (c) percentage weight change measured from before DSS exposure (day 4) to necropsy (day 12); (d) representative images (200x magnification) of colon H and E sections; (e) histology scores based on inflammatory cell infiltration, goblet cell loss, mucosal architecture, muscle thickening, edema, and crypt abscess; (f) heatmap of relative gene expression changes in the colon of DSS challenged mice during 2ʹFL supplementation; and (g) serum levels of LPS binding protein (LBP). In (a) treatments consisted of 5 groups of mice: untreated = oral gavage of PBS (day 1–4) and drinking water (day 1–12); DSS = oral gavage of PBS (day 1–4) and DSS in drinking water (day 5–9); DSS + *B.p*. MP80 = oral gavage of *B.p*. MP80 (day 1–4) and DSS in drinking water (day 5–9); DSS + 2ʹFL = oral gavage of PBS (day 1–4), DSS in drinking water (day 5–9), and 2ʹFL in drinking water (day 1–12); DSS + *B.p*. MP80 + 2ʹFL = oral gavage of *B.p*. MP80 (day 1–4), DSS in drinking water (day 5–9), and 2ʹFL in drinking water (day 1–12); *n* = 6 per treatment group. In (c), (e), and (g) the different letters signify statistical difference between treatments (one-way ANOVA with FDR correction; *p* < .05). In (B) outliers were excluded using Grubb’s test (α = .05). In (f) Log_2_ transformed fold changes are expressed with DSS alone challenged mice values expressed relative to untreated control mice and all other DSS-challenged mice expressed relative to DSS alone challenged mice (statistical analysis contained in Supplemental fFgure S7 and Supplemental Tables S18-S19). Genes were grouped according to known function: serotonin (5-HT) regulatory targets, aryl hydrocarbon receptor (AhR) pathway targets, intestinal barrier markers, anti-inflammatory, pro-inflammatory, inflammatory modulating, and inflammatory metabolism

The DSS-induced colitis decrease in body weight was significantly attenuated by treatment with *B.p*. MP80 + 2ʹFL (one-way ANOVA with FDR correction, *p* < .0001, DSS vs untreated; *p* = .0004, DSS vs DSS + *B.p* MP80 + 2ʹFL; Figure 4a-c) and significantly attenuated by pretreatment with 2ʹFL alone (*p* = .005, DSS vs DSS + 2ʹFL; Figure 4c, Supplemental Table S17). There was no significant difference in water intake between any groups throughout the experimental period, indicating that all groups received the same amount of DSS (Supplemental Table S17). DSS-induced immune cell infiltration, increase in colon length, disrupted mucosal architecture, and muscle thickening in the colon were significantly attenuated by *B.p*. MP80 + 2ʹFL treatment (one-way ANOVA with FDR correction, *p* = .0001, DSS vs untreated; *p* = .005, DSS vs DSS + *B.p* MP80 + 2ʹFL; Figure 4d-e;), but not by *B.p*. MP80 or 2ʹFL treatments alone (*p* = .90, DSS vs DSS + *B.p* MP80; *p* = .82, DSS vs DSS + 2ʹFL). Expression of occludin was significantly reduced by DSS compared to untreated (Wilcoxon Rank Sum with FDR correction, *p* = .024; Figure 4f, Supplemental Table S19); treatment with *B.p*. MP80 + 2ʹFL significantly attenuated this reduction (*p* = .75, untreated vs DSS + *B.p* MP80 + 2ʹFL; *p* = .05, DSS vs DSS + *B.p* MP80 + 2ʹFL; Supplemental Tables S18-S21). Impairment of intestinal barrier function was assessed by measuring plasma levels of LPS binding protein (LBP); plasma LBP was significantly increased in mice with DSS (*p* < .0001, untreated vs DSS) which was significantly reduced by *B.p*. MP80 + 2ʹFL treatment (one-way ANOVA with FDR correction, *p* = .42, untreated vs DSS + *B.p* MP80 + 2ʹFL; *p* < .0001 DSS vs DSS + *B.p* MP80 + 2ʹFL; Figure 4g).

DSS significantly increased the expression of inflammatory markers IL-1β, IL-6, and Ccl2 and decreased expression of a number of anti-inflammatory pathways including Tgfβ and AhR in the colon compared to controls (Wilcoxon Rank Sum with FDR correction, *p* = .024 for each gene respectively; Supplemental Figure S7; Supplemental Tables S18-S19). *B.p*. MP80 + 2ʹFL pre-treatment prevented the DSS-induced increases in IL-6 and Ccl2 (*p* = .024, respectively). Similar trends were observed in the liver where *B.p*. MP80 + 2ʹFL treatment attenuated DSS-induced increase in Lcn and decrease in Tgfβ (*p* = .013, *p* = .020, DSS vs DSS + *B.p* MP80 + 2ʹFL; Supplemental Figure S9, Supplemental Tables S22-S23). Commensal microbes such as *Lactobacillus* and *Bifidobacterium spp*. produce agonists that activate the aryl hydrocarbon receptor (AhR) pathway shown to be involved in intestinal homeostasis.^30^ Specifically, *Bifidobacterium* produce indole metabolites such as indole-3-lactate which is an agonist for the AhR and interacts with the serotonin reuptake transporter, Slc6a4.^31,32^ Decreases in Slc6a4 are associated with colitis whereby activation of AhR is associated with improved disease outcomes.^33^ Here we show that *B.p*. MP80 + 2ʹFL prevented the colonic DSS-induced decrease in AhR (*p* = .036, DSS vs DSS + *B.p* MP80 + 2ʹFL; Figure 4f, Supplemental Tables S18-S19). The decrease in serum LBP and increase in AhR and colon length were significantly correlated to *B.p*. MP80 qPCR numbers (Spearman’s correlation, *p* = .008, *p* = .004, *p* = .001, Supplemental Figure S11, Supplemental Table S24). When administered simultaneously the data show that the effect of *B.p*. MP80 + 2ʹFL treatment on DSS-colitis was significantly greater than treatment with either *B.p*. MP80 or 2ʹFL alone.

Pre-treatment with *B.p*. MP80 + 2ʹFL shifted serum metabolite profiles to be more consistent with profiles of untreated mice in comparison to DSS mice (Supplemental Figure S12a). Treatment effects (Hedge’s g, |g| > .5) showed an increase in the serum metabolites glucose, 3-hydroxybutyrate, acetate and formate in DSS + *B.p*. MP80 + 2ʹFL mice versus mice receiving DSS alone (Supplemental Figure S12b). Conversely, DSS mice had elevated branch chain amino acids (leucine, isoleucine and valine) in addition to lysine, phenylalanine and pyruvate (Hedge’s g, |g| > .5) relative to DSS + *B.p*. MP80 + 2ʹFL mice (Supplemental Figure S12 c).

### Attenuated neuroinflammation after treatment with B.p. MP80 and 2ʹFL

Systemic peripheral inflammation has been reported to induce neuroinflammation, therefore, we assessed brain samples from DSS-challenged animals to determine severity in this model and the effects of *B.p*. MP80 + 2ʹFL treatment.^34^ DSS induced pro-inflammatory cytokine Cxcl1 expression in the hypothalamus (Wilcoxon Rank Sum with FDR correction, *p* = .014; Supplemental Figure S13). Cxcl1 induction was prevented in the hypothalamus by *B.p*. MP80 + 2ʹFL treatment (*p* = .014). This effect on Cxcl1 suggests further investigation is needed.

## Discussion

Numerous clinical trials of probiotic administration have failed to show significant impact on the host microbiota partly because engraftment of a supplemented microbe into a stable microbial community has proven challenging.^20,35^ One strategy for enriching probiotics *in situ* is to provide a unique substrate preferentially catabolized by the supplemented bacteria.^1^ Such synbiotic pairings have been shown to elevate the microbe’s abundance in the gut during simultaneous administration, however, detection of the microbe post-bacterial gavage (i.e. beyond supplementation) is not typically measured.^36,37^ Bacterial persistence was demonstrated with *Bacteroides* strains engrafting in mice microbiota dependent upon the presence of porphyran as a substrate for the supplemented strains.^7,8^ Similarly, in breast-fed infants high *Bifidobacterium* levels are associated with the ability to catabolize HMOs.^23^ In preterm infants supplemented with *Lactobacillus* and *Bifidobacterium*, only *Bifidobacterium* robustly persisted and was correlated to milk metabolism.^38^ Frese et al.^39^ showed dramatic and persistent colonization of an HMO-catabolizing strain of *Bifidobacterium longum* subsp. *infantis* in supplemented breast-fed infants. This is in concordance with the findings presented here that, in the absence of other human milk factors, the milk glycan 2ʹFL is sufficient to enable persistence and enrich populations of cognate *Bifidobacterium* while competing with endogenous bacterial groups.

Our data show that 2ʹFL, an HMO nutrient resource exogenous to the mouse intestine, provides a fitness advantage to *B. pseudocatenulatum* that possess 2ʹFL catabolism genes. In the presence of 2ʹFL, *B.p*. MP80 persisted robustly while *B.p*. JCM11661 failed, suggesting the fucosylated HMO gene cluster is required for persistence. Degradation of 2ʹFL was detected by the fucose catabolism byproduct 1,2-propanediol, of which *B.p*. MP80 is known to produce.^26,^^40^ 1,2-propanediol is a differentiating serum marker between breast-fed and formula fed infants linked to *Bifidobacterium* metabolism.^41^ Here, high fecal *B.p*. MP80 is associated with 1,2-propanediol in the colon contents suggesting that 2ʹFL is being catabolized by *B.p*. MP80 via fucose catabolism. This catabolism provides a growth advantage that enables persistence and consequently ensures active metabolism that may be connected to host benefits.

Synbiotic treatment with *B.p*. MP80 and 2ʹFL is associated with a reduction in α-diversity (Shannon Index) in contrast to *B.p*. JCM11661. Thus, *B.p*. MP80 in the presence of 2ʹFL successfully reduced the richness and evenness of the endogenous microbial community species (Shannon Index) which are associated with colonization resistance.^4^ These results are emblematic of the diversity-invasion effect, where survival of a microbial invader is negatively associated with species richness and evenness.^4^

The microbial community, as measured by β-diversity and differential abundance, was restructured by 2ʹFL supplementation alone. 2ʹFL is likely more accessible to a murine endogenous microbiota than previously studied substrates.^7,8^ Given HMOs resemble mucin glycans, mouse endogenous *Bacteroidaceae* and *Bifidobacteriaceae* likely possess α-fucosidases capable of cleaving 2ʹFL. 2ʹFL enrichment of *Bacteroidaceae* and *Bifidobacteriaceae* implies that an invading *Bifidobacterium* must outcompete these endogenous microbes for 2ʹFL and the persistence of *B.p* MP80 suggests that it is a strong competitor. Further research is warranted to investigate how glycan specificity, host microbial barriers, and microbial genetic capabilities influence *Bifidobacterium* fitness in a more competitive environment, such as the mature human gut, and whether a more selective nutrient resource would increase fitness of cognate *Bifidobacterium* strains.

*Bifidobacterium* supplementation has previously been associated with reduced DSS-induced colitis inflammation although effects seem to be strain-specific.^42^ In prior synbiotic experiments continuous gavage of *Bifidobacterium* was required for colitis amelioration.^43^ Here we show *B.p*. MP80 persistence maintained by the presence of 2ʹFL significantly reduces the severity of DSS-induced colitis, an example of bacterial-carbohydrate synergy impacting host physiology. The ability of *B.p*. MP80 in the presence of 2ʹFL to reduce colitis severity is in marked contrast to the lack of effect of *B.p*. MP80 alone. *B.p*. MP80 + 2ʹFL was associated with decreased expression of pro-inflammatory cytokines IL-6, IL1-β and CCl2, previously induced in colitis models and with increased expression of Tgfβ.^44^ Tgfβ administration is associated with improved health outcomes while anti-Tgfβ worsened outcomes.^45,46^ Consistent with reports of *Bifidobacterium* preserving intestinal barrier function, *B.p*. MP80 in the presence of 2ʹFL increased occludin expression and decreased plasma LBP levels.^42,47^ The increase in Tgfβ and occludin positively correlate with 2ʹFL-dependent *B.p*. MP80 abundance.

Prior *in vitro* experiments identified *Bifidobacterium*-produced metabolite indole-3-lactic acid (ILA) acts upon AhR and Nrf2 pathways.^16^ The transcription factor hypoxia inducible factor (HIF) can be activated through cytoprotective AhR and Nrf2 pathways, creating a hypoxic environment which plays a role in mucosal protection *in vivo*.^48,49^ There was increased expression of AhR in the colon of DSS + *B.p*. MP80 + 2ʹFL treated mice suggesting activation of this pathway. However, further research is required to identify specific metabolites that are activating the AhR, Nrf2, and serotonin pathways *in vivo*.

Glucose, 3-hydroxybutyrate, acetate and formate were higher in the serum of DSS + *B.p*. MP80 + 2ʹFL mice relative to DSS mice. Notably, breast-fed infant serum metabolites are elevated in acetate and formate.^41^ *B.p*. MP80 *in vitro* growth on 2ʹFL produces acetate and formate as major fermentation products. Increased acetate concentrations are associated with higher *Bifidobacterium* abundance and is shown to provide a protective effect during inflammatory challenge of mice.^15,50^ Acetate is absorbed in the cecum and colon and is subsequently detected in venous blood.^51–54^ Conversely, DSS mice exhibited decreases in serum glucose and 3-hydroxybutyrate alongside increased branch chain amino acids relative to DSS + *B.p*. MP80 + 2ʹFL treated mice. Others have suggested this metabolic imbalance is likely indicative of tissue catabolism to rectify the loss of energy intake through diet in DSS challenged mice.^55^

2ʹFL treatment alone has some beneficial effects in DSS-induced colitis, including attenuation of body weight change and reduced serum LBP although there was no effect on colon histology scores or spleen weight. 2ʹFL has been shown to reduce systemic inflammation in mice.^56^ 2ʹFL is likely acting via both direct impact on host cells and indirect catabolism of 2ʹFL by endogenous microbes, including *Bifidobacterium*, to produce bioactive metabolites. This is supported by 2ʹFL alone activating similar pathways, including AhR/Nrf2 target genes Gpx2, Nqo1, and Hmox1 in the cecum. 2ʹFL alone reduces inflammation, however the provision of an anti-inflammatory *Bifidobacterium* with a HMO has a synergistic protective effect, noticeably improving health outcomes when compared to *B.p*. MP80 or 2ʹFL alone.

It is challenging to predict how amenable a mature microbiota is to microbial engraftment, in this current study *B.p*. MP80 paired with 2ʹFL consistently persisted in multiple cohorts and bacterial abundance was correlated to the concentration of 2ʹFL provided to the mice. We conclude that by providing a bacterial-carbohydrate pairing that is biologically relevant and evolutionarily selected, we improved the likelihood of engraftment and associated beneficial health outcomes in mice. This concept is directly applicable toward development of synbiotic pairings for other live bacterial therapeutics targeting mature microbiotas.

At present, the specific role of HMOs in the neonatal enrichment of a *Bifidobacterium* population is solely associative as there are multiple factors in milk known to shape the infant gut microbiome.^57,58^ However, the data presented here show a single HMO (2ʹFL) is able to promote enrichment of a cognate 2ʹFL-consuming *Bifidobacterium* strain within a complex gut ecosystem. This argues that HMOs alone are sufficient to drive this outcome and provides a novel model to examine the specific influences of HMO-*Bifidobacterium* axis in isolation. While disparities between our mouse model and the human intestine may limit our conclusions, they also provide future research questions. The established mouse microbial community is not characteristic of the naïve infant’s microbial community structure or colonization resistance. The infant gut initially possesses fewer bacterial species, however, those bacteria are being selected based on their capacity to catabolize nutrients found in breast milk. Therefore, the impact of founder effect, bacterial fitness differences, and inter-species competition of *Bifidobacterium* strains should be investigated in the future.

This research suggests that HMOs act as a privileged nutrient resource, enriching bacterium capable of catabolism even when high colonization resistance is present. This is an important concept for clinicians when addressing infant gastrointestinal microbiota development and adult GI inflammatory diseases. It provides critical information on bacterial characteristics that should be considered when recommending probiotics or live bacterial therapeutics and may increase the likelihood of conferring health benefits. Furthermore, these findings demonstrate the critical role of HMOs in colonization of *Bifidobacterium* which is associated with lifelong health impacts for infants and supports current efforts to encourage breast feeding.

## Methods

### Mouse studies

Animals were handled and maintained in accordance with protocols approved by the Institutional Animal Care and Use Committee of University of California-Davis (IACUC Protocol: 21900). Male C57BL/6 J mice (5–6 weeks old, Jackson Labs) were group housed (3 per cage) and maintained at 22°C with 12-hour light-dark cycle. Before commencing experiments, mice were co-housed and acclimated for a minimum of 1 week at the facility. Food (5058 Irradiated Pico Mouse Lab Diet) and water were provided *ad libitum*. 2ʹFL (Advanced Protein Technologies Corporation, Korea) was provided in the drinking water as a 10% (w/v) solution. *Bifidobacterium* (10e9 cfu/ml in PBS) or phosphate buffered saline was administered via oral gavage (100 µl). Mice were euthanized via CO_2_ asphyxiation, excluding DSS experiments where mice were euthanized with FatalPlus. Supplemental methods contain further details on experimental design, preparation of bacterial inoculum and 2ʹFL, and ^1^H-NMR metabolite sample preparation and analysis.

### Quantification of bacterial strains by qPCR

*B.p*. JCM11661 was quantified with strain-specific primers designed for this study while *B.p*. MP80 and *Bifidobacterium* primers were previously generated (Supplemental table S1).^35,59,60^ Primer validation and PCR program located in supplemental methods.

### Fecal extraction, microbiota DNA sequencing, and differential abundance testing

Fecal samples were collected from individual mice within 1 hour of the light cycle’s start. DNA was extracted from 30 to 100 mg of stool sample using the Quick-DNA Fecal/Soil Microbe Miniprep Kit, Catalog No. D6010 (ZYMO, Irvine, CA, USA). The extraction protocol was in accordance with the manufacturer’s instructions including a bead-beating step using a FastPrep-24 Instrument (MP Biomedicals, Santa Ana, CA, USA) for a total of 2 min at 25°C at a speed of 6.5 m/s. In triplicate, the V4 region of the *16S rRNA* gene was amplified with barcoded PCR primers F515 (5′-CACGGTCGKCGGCGCCATT-3′) and R806 (5-′GGACTACHVGGGTWTCTAAT-3′) modified to contain an adapter region for sequencing on the Illumina MiSeq platform.^61^ Amplicons were verified by gel electrophoresis, combined, purified, and sent to the UC Davis Genome Center for library preparation and high throughput 250-bp paired-end sequencing using the Illumina MiSeq platform. Raw sequencing data was demultiplexed and quality filtered before import into QIIME2-2019.7.^62^ Samples with poor quality data were excluded from analysis. After trimming, reads were processed with DADA2.^63^ Filtered sequences were aligned and taxonomy was assigned using the 99% SILVA naïve Bayesian classifier in QIIME 2 v2019.7.^64^ Samples were rarified to 2000 sequences. Differential abundance was evaluated with Songbird which ranks the log-fold changes between selected taxa or ASVs, identifying ASVs as high or low ranked.^29^ The Songbird formula for differential abundance testing between all treatments tested the interaction between *B.p*. MP80 and 2ʹFL while accounting for the longitudinal nature of data. For 2ʹFL differential abundance, mice supplemented with a *Bifidobacterium* were excluded from analysis and only 2ʹFL and PBS treatments were evaluated. Taxa *Bifidobaceriaceae* and *Bacteroidaceae* were chosen as the numerator for respective analyses based on high and low Songbird rankings. For both respective analyses, the lowest 25% of ranked ASVs were selected and *Lachnospiraceae* and *Ruminococcaceae* ASVs identified within that selected range were chosen as the denominator when calculating log-fold changes. Supplemental Tables S6 and S7 list ASVs used for each log ratio. The NCBI BioProject ID for raw *16S* sequencing data is PRJNA669815. Analysis of microbial ecosystem characteristics and statistics located in supplemental methods.

### Plasma and tissue collection

Blood was collected via cardiac puncture into EDTA-coated vaccutainers. After centrifugation (40°C, 10,000 RCF, 15 min), plasma was obtained and stored at −80°C. Luminal contents and tissue from small and large intestine, liver, and brain were collected onto dry ice before storage at −80°C.

### Barrier function assessment

GI tract was cut along the mesenteric border and mounted in Ussing chambers inserts exposing 0.1 cm^2^ tissue surface area (Physiologic Instruments, San Diego, CA, USA). The mucosal tissue side was exposed to a Ringers-mannitol (10 mM) solution and the serosal was exposed to a Ringers-glucose (10 mM) solution. Both compartments were oxygenated, and tissue maintained at 37°C. To measure paracellular and transcellular permeability, FITC-labeled dextran (400ug/ml, FD-4, Sigma Aldrich) and horse radish peroxidase (200ug/ml, HRP Type VI, Sigma Aldrich), respectively, were added to the mucosal compartment. Every 30 minutes for the next 2 hours, serosal samples were collected. Concentration of FD-4 was measured with fluorescence (485 nm excitation, 538 nm emission) whereas HRP was detected by O-dianisidine (450 nm absorbance). Data was calculated as flux (ng/cm2/hr).

### Plasma lipopolysaccharide-binding protein (LBP)

LBP were measured in plasma samples via ELISA as per manufacturer instructions (Biometec GmbH, Greifswald, Germany).

### Histology

Colon sections from DSS-treated mice were embedded in paraffin and cut into 10 µM sections, mounted on slide and processed for hematoxylin and eosin staining. Images were taken at 200X using the MetaMorph Basic v. 7.7.0 image-analyzer software on an Olympus BX61 microscope. Tissues were scored blindly from 3 sections from every mouse on a scale of 1–4 based on inflammatory cell infiltration, goblet cell loss, mucosal architecture, muscle thickening, edema, and crypt abscess as previously described.^65,66^

### RNA extraction and qRT-PCR

RNA was extracted from all intestine sections, liver, and brain using the TRIzol method (Life technologies, 15596018). Quality and quantity of RNA was assessed using a NanoDrop Spectrophotometer (Thermo Scientific). cDNA synthesis was performed (1ug RNA) with iScript cDNA synthesis kit (BioRad, 1708890) (primer sequences in Supplemental table S6). Real-time PCR was performed using Quantstudio 6 Flex real-time PCR machine with PowerUp SYBR Green Master Mix (Thermo Fisher, A25742) for detection. Ribosomal protein L13a was used as a housekeeping gene in accordance to the 2^ΔΔCT^ method. Gene expression data are normalized to untreated control mice. In the heat maps, measured genes are expressed as the Log_2_ transformed fold change in mRNA expression levels relative to the untreated group.

### Colitis model statistics

Data are expressed as means ± SEM and are analyzed by Kruskal-Wallis test with FDR correction and post hoc Wilcoxon Rank Sum test with FDR correction (*p* < .05 as significant). Outliers excluded based on Grubbs’ test α = .05. Spearman’s test calculated correlations between DSS inflammatory measures (qRT-PCR) and *B.p*. MP80 abundance (qPCR) (Supplemental Table S24).

## Supplementary Material

Supplemental Material

## Data Availability

The NCBI BioProject ID for raw *16S* sequencing data is PRJNA669815.
