# Supplementary figures and images for: Bifidobacterium catabolism of human milk oligosaccharides overrides endogenous competitive exclusion driving colonization and protection
Source: Gut Microbes. 2021 Oct 27;13(1):1986666. doi: 10.1080/19490976.2021.1986666 (PMC8555557; doi:10.1080/19490976.2021.1986666)

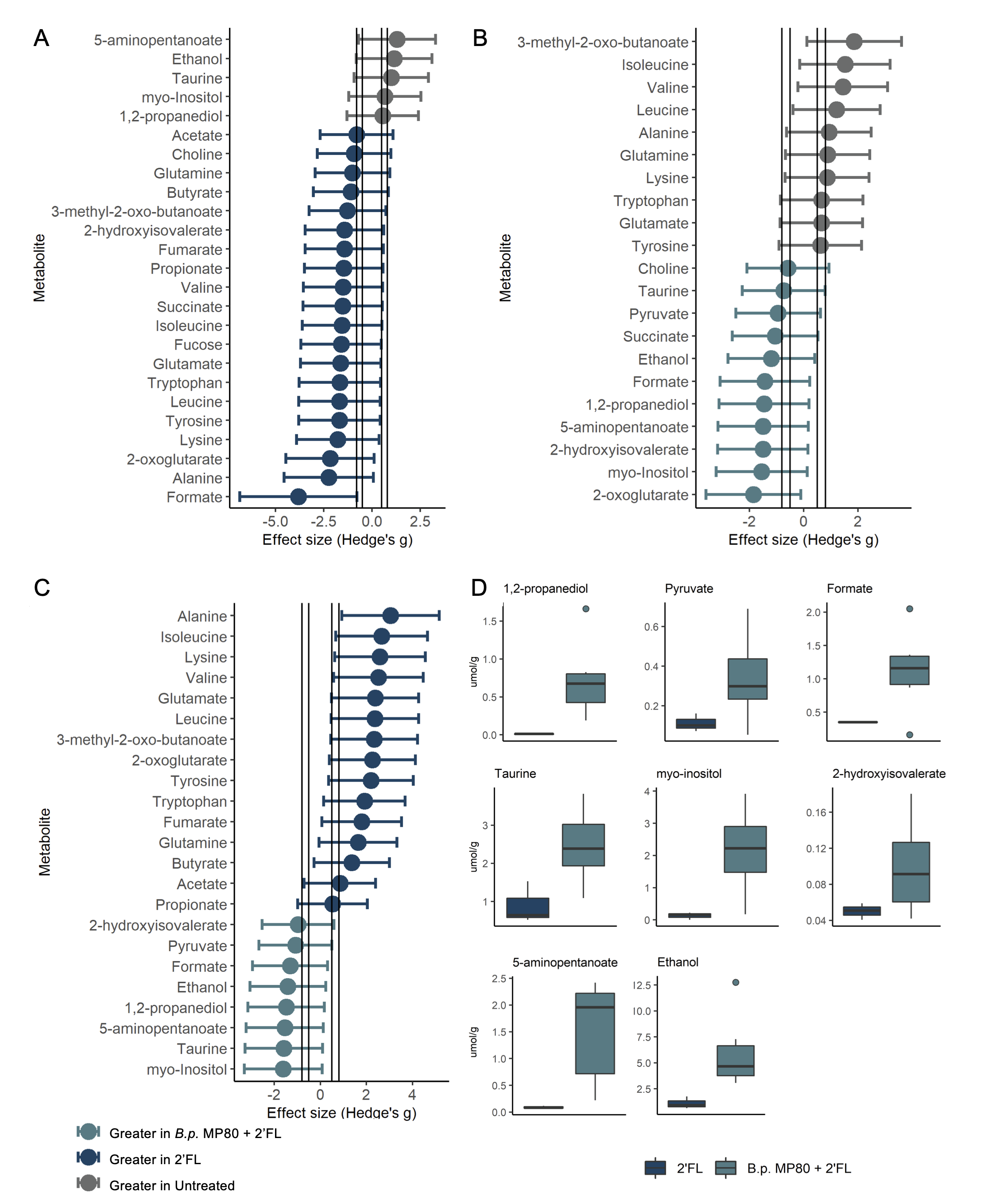

Supplement: Supplemental Material [file KGMI_A_1986666_SM4160.zip › Supplementary information/SuppFig1.tiff]

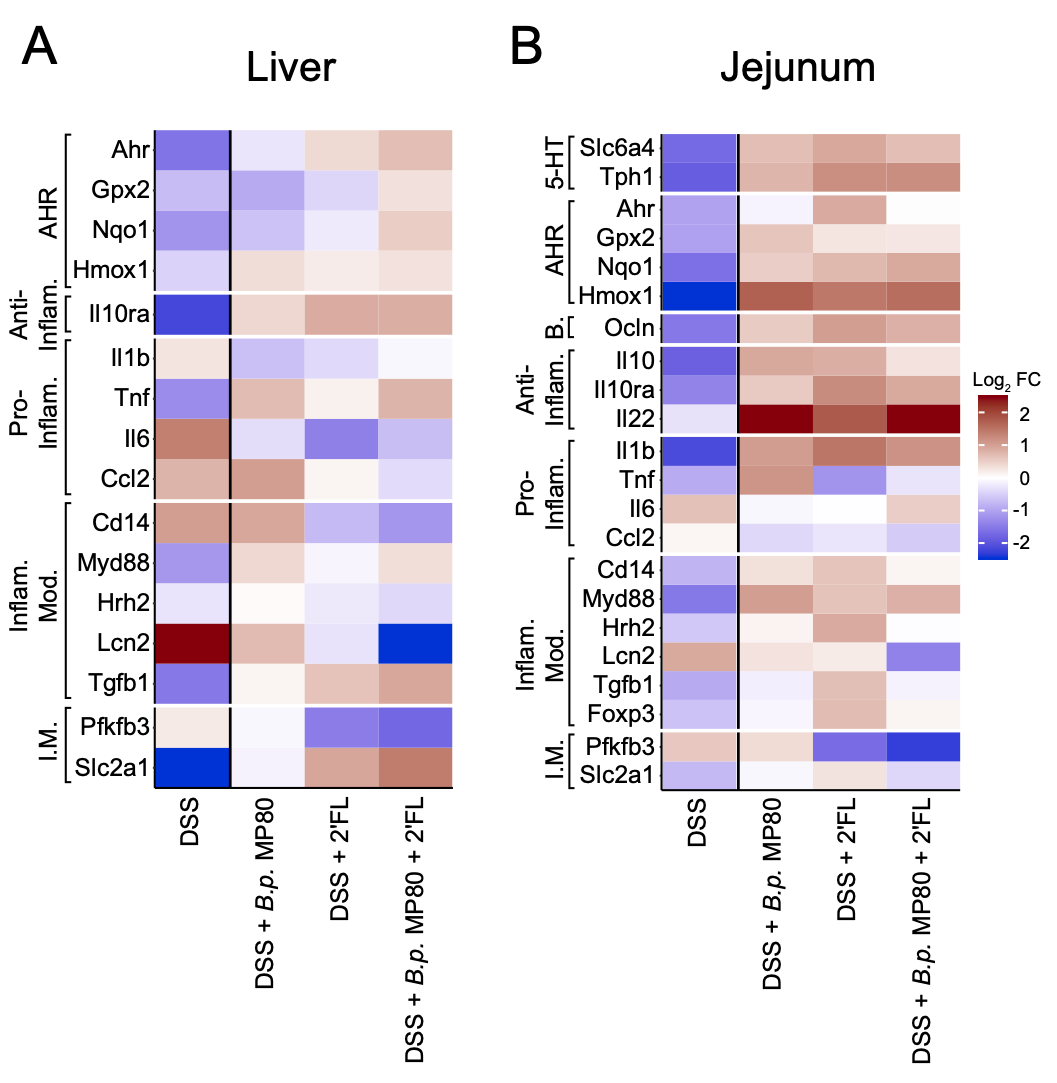

Supplement: Supplemental Material [file KGMI_A_1986666_SM4160.zip › Supplementary information/SuppFig10.tiff]

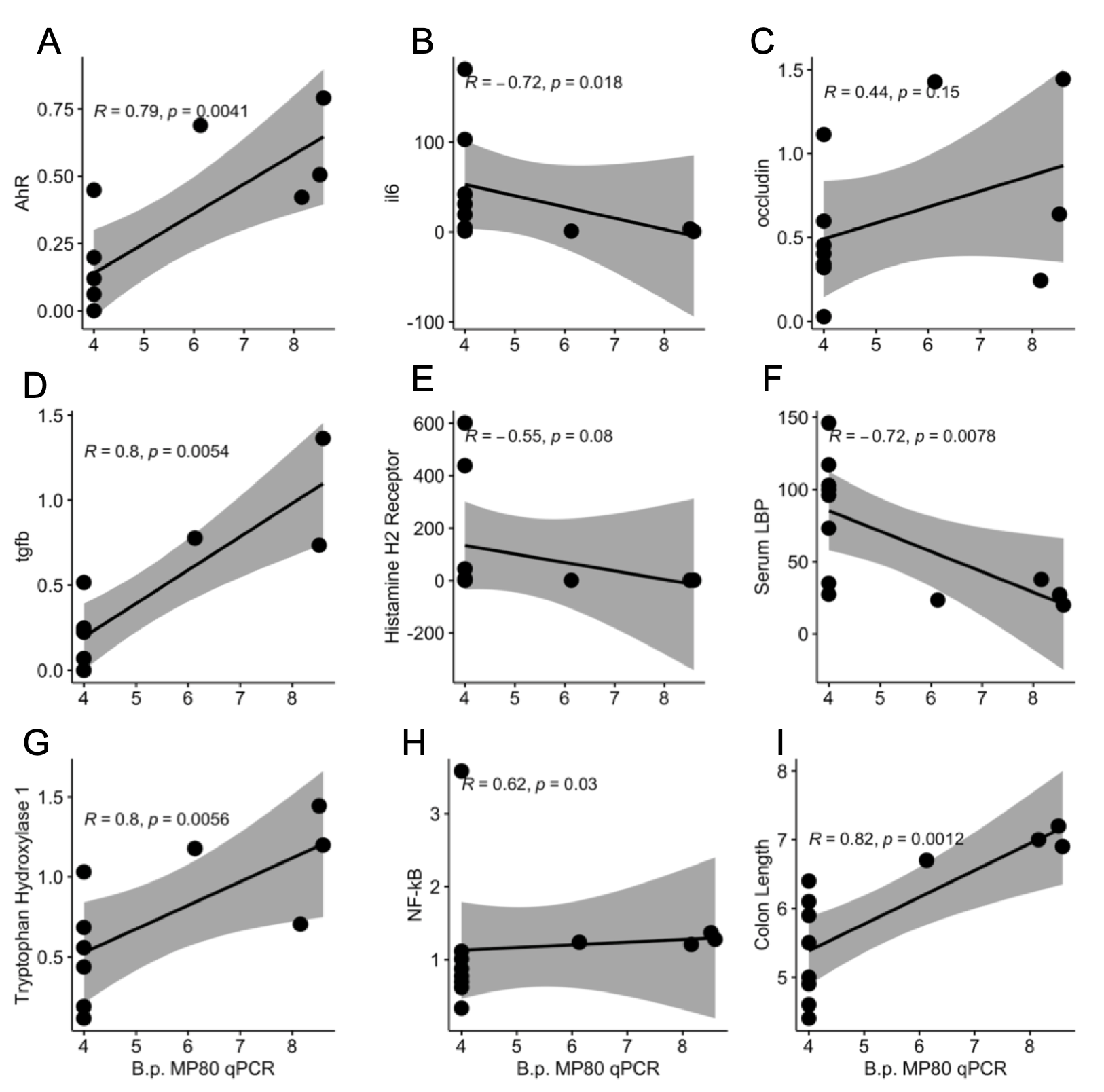

Supplement: Supplemental Material [file KGMI_A_1986666_SM4160.zip › Supplementary information/SuppFig11.tiff]

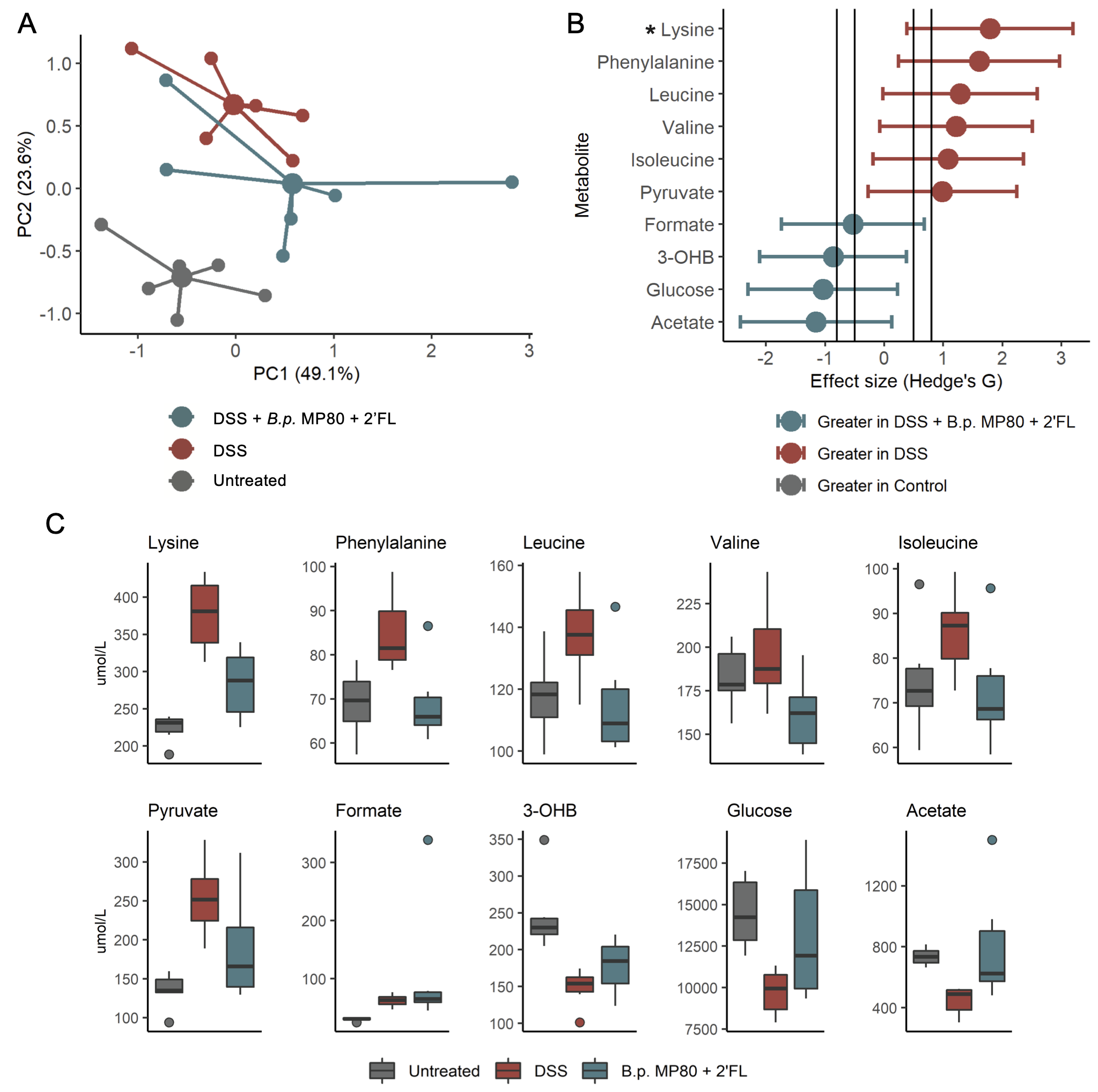

Supplement: Supplemental Material [file KGMI_A_1986666_SM4160.zip › Supplementary information/SuppFig12.tiff]

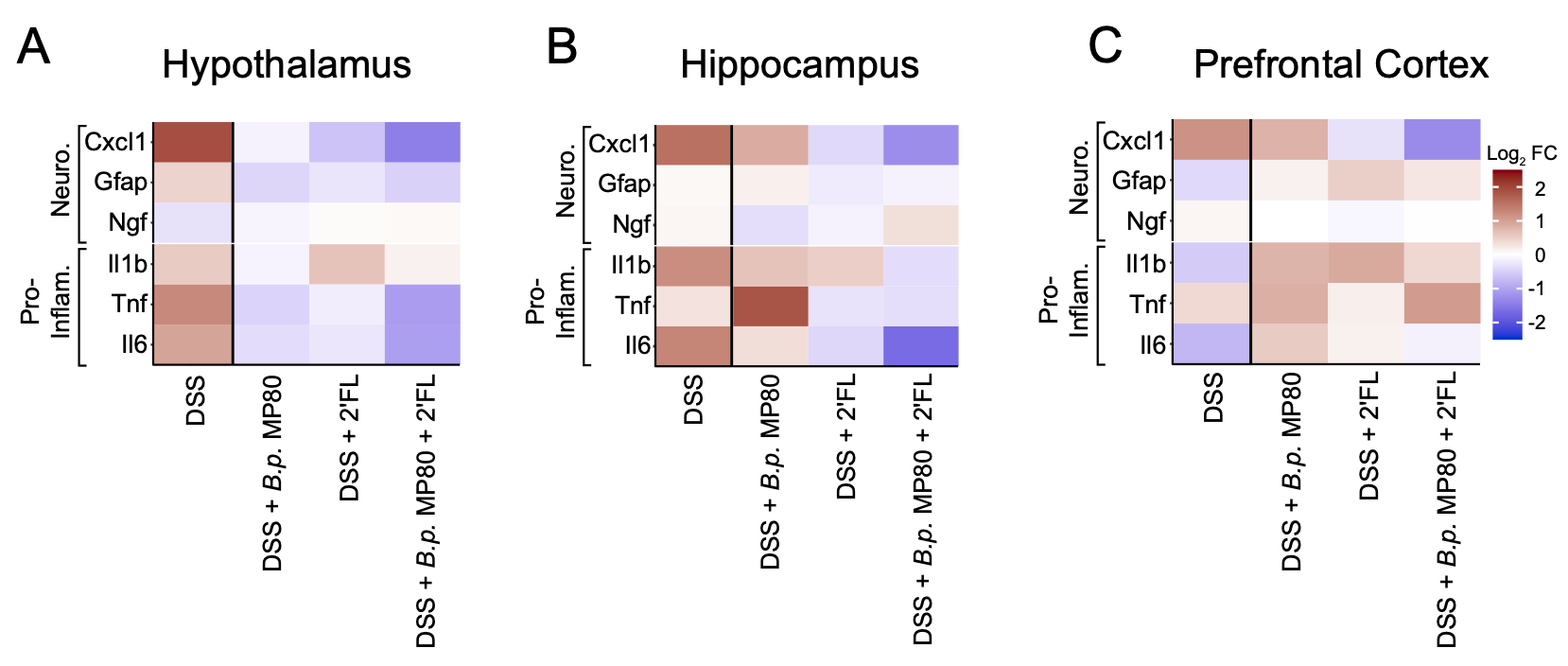

Supplement: Supplemental Material [file KGMI_A_1986666_SM4160.zip › Supplementary information/SuppFig13.tiff]

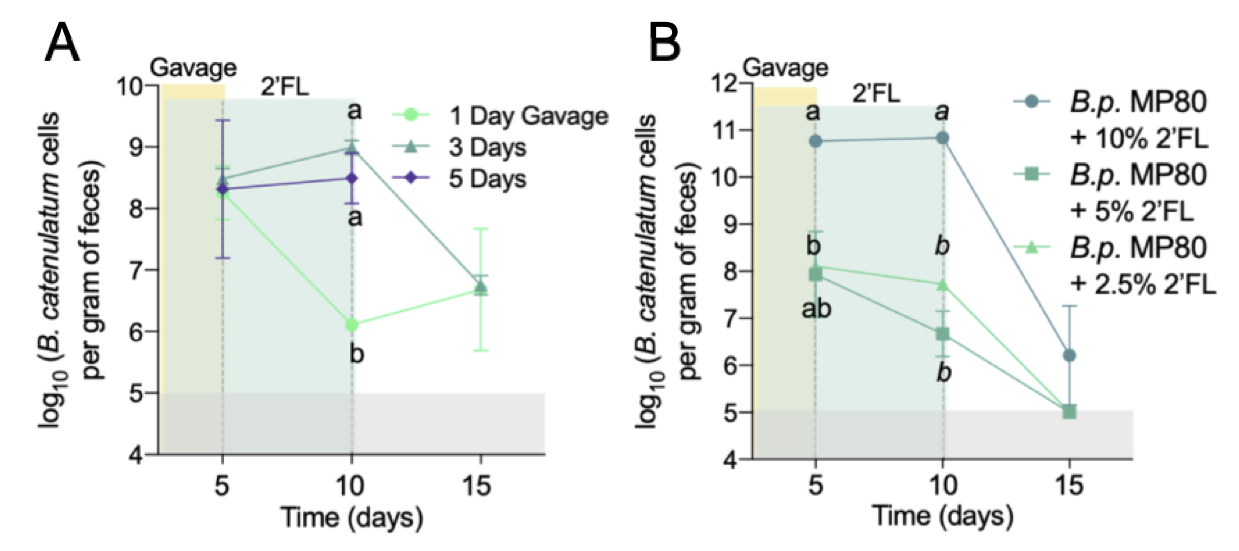

Supplement: Supplemental Material [file KGMI_A_1986666_SM4160.zip › Supplementary information/SuppFig2.tiff]

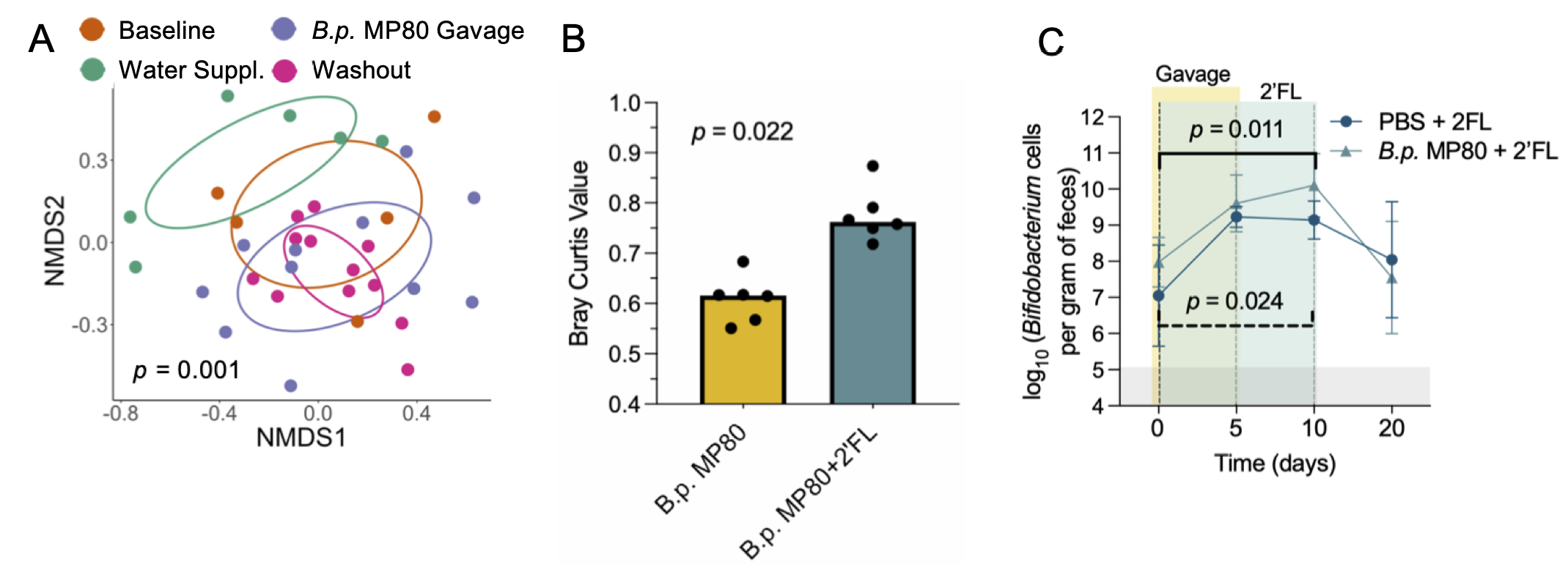

Supplement: Supplemental Material [file KGMI_A_1986666_SM4160.zip › Supplementary information/SuppFig3.tiff]

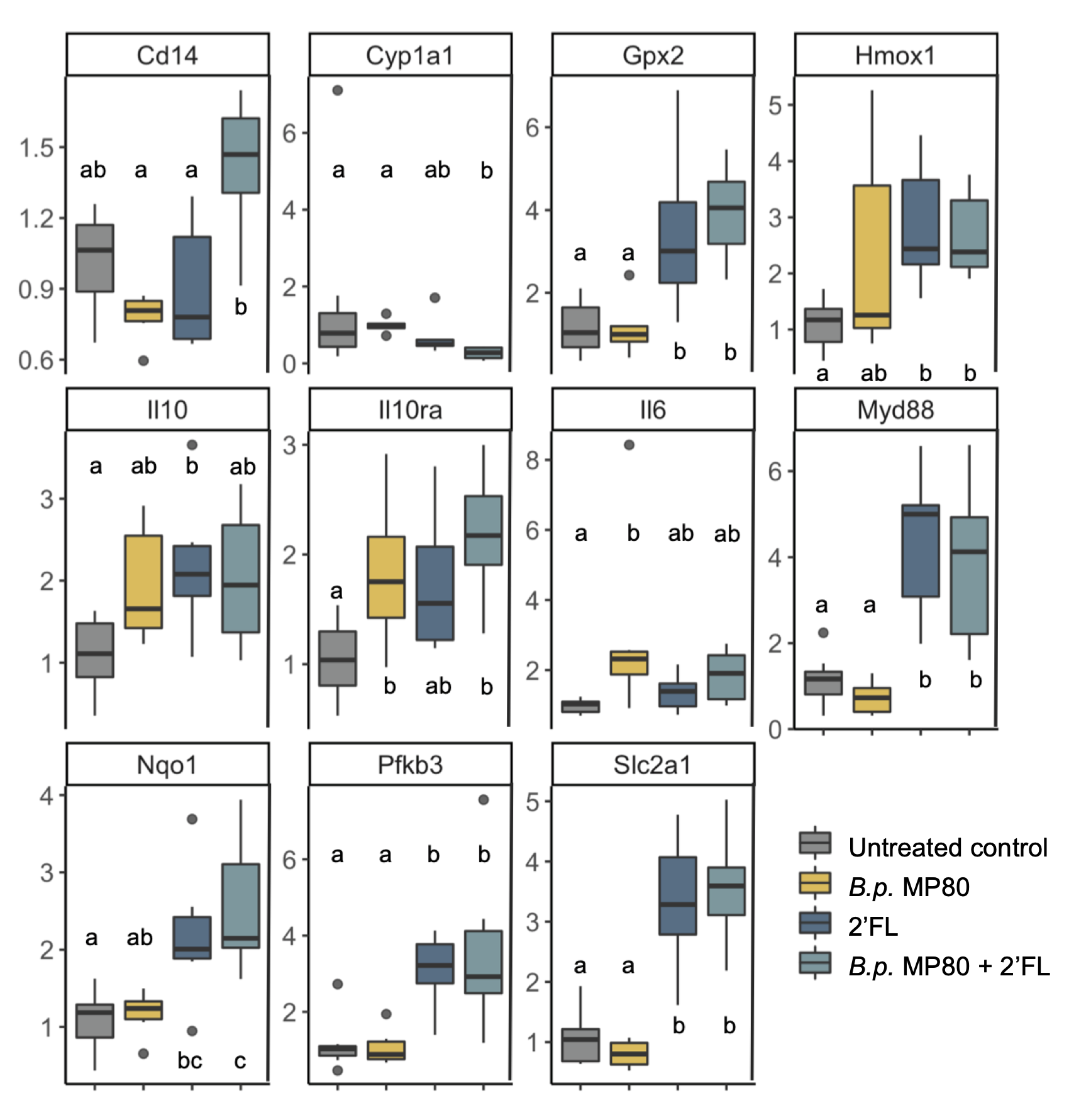

Supplement: Supplemental Material [file KGMI_A_1986666_SM4160.zip › Supplementary information/SuppFig4.tiff]

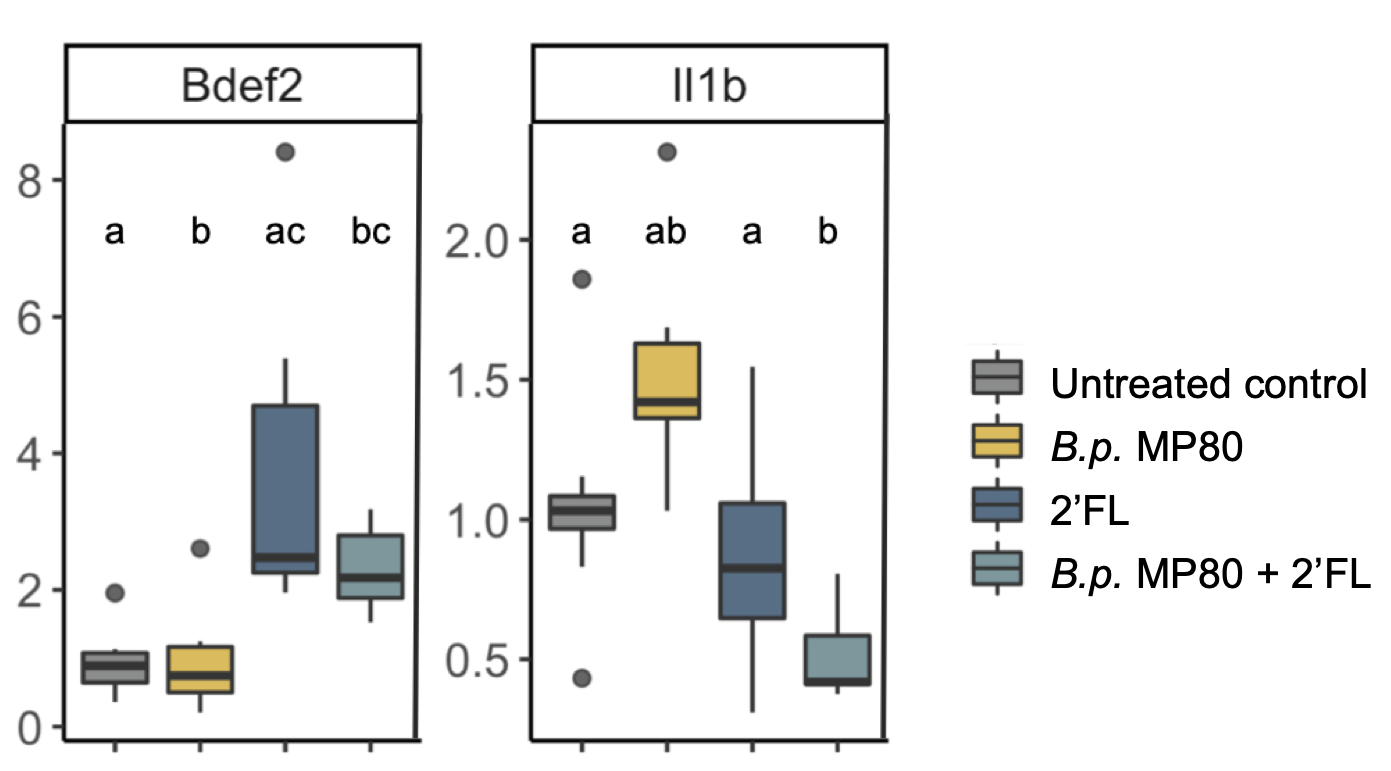

Supplement: Supplemental Material [file KGMI_A_1986666_SM4160.zip › Supplementary information/SuppFig5.tiff]

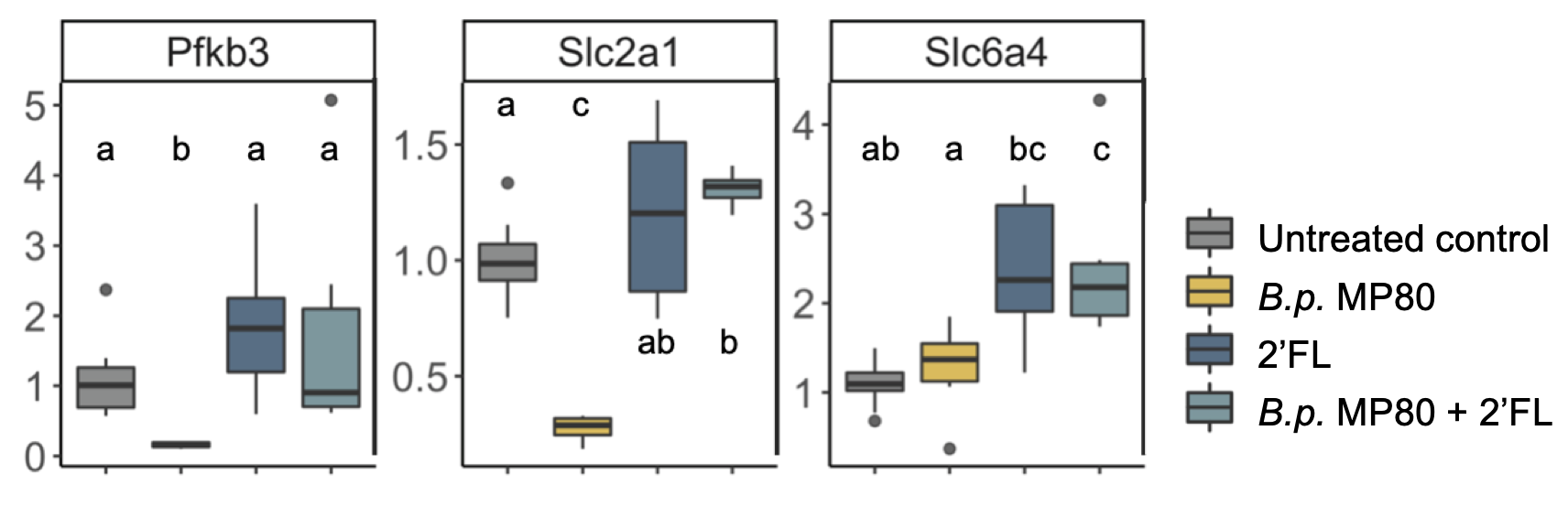

Supplement: Supplemental Material [file KGMI_A_1986666_SM4160.zip › Supplementary information/SuppFig6.tiff]

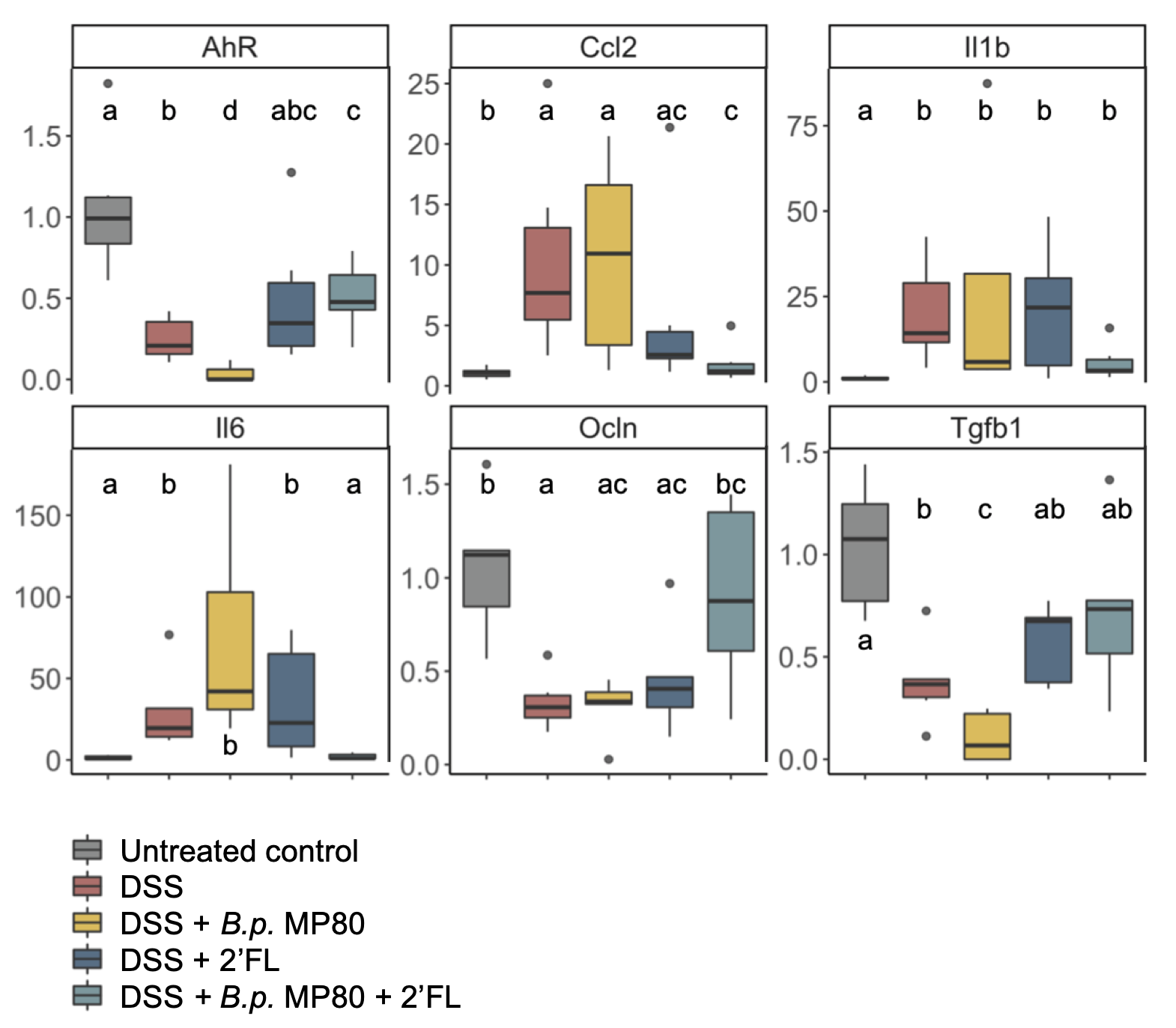

Supplement: Supplemental Material [file KGMI_A_1986666_SM4160.zip › Supplementary information/SuppFig7.tiff]

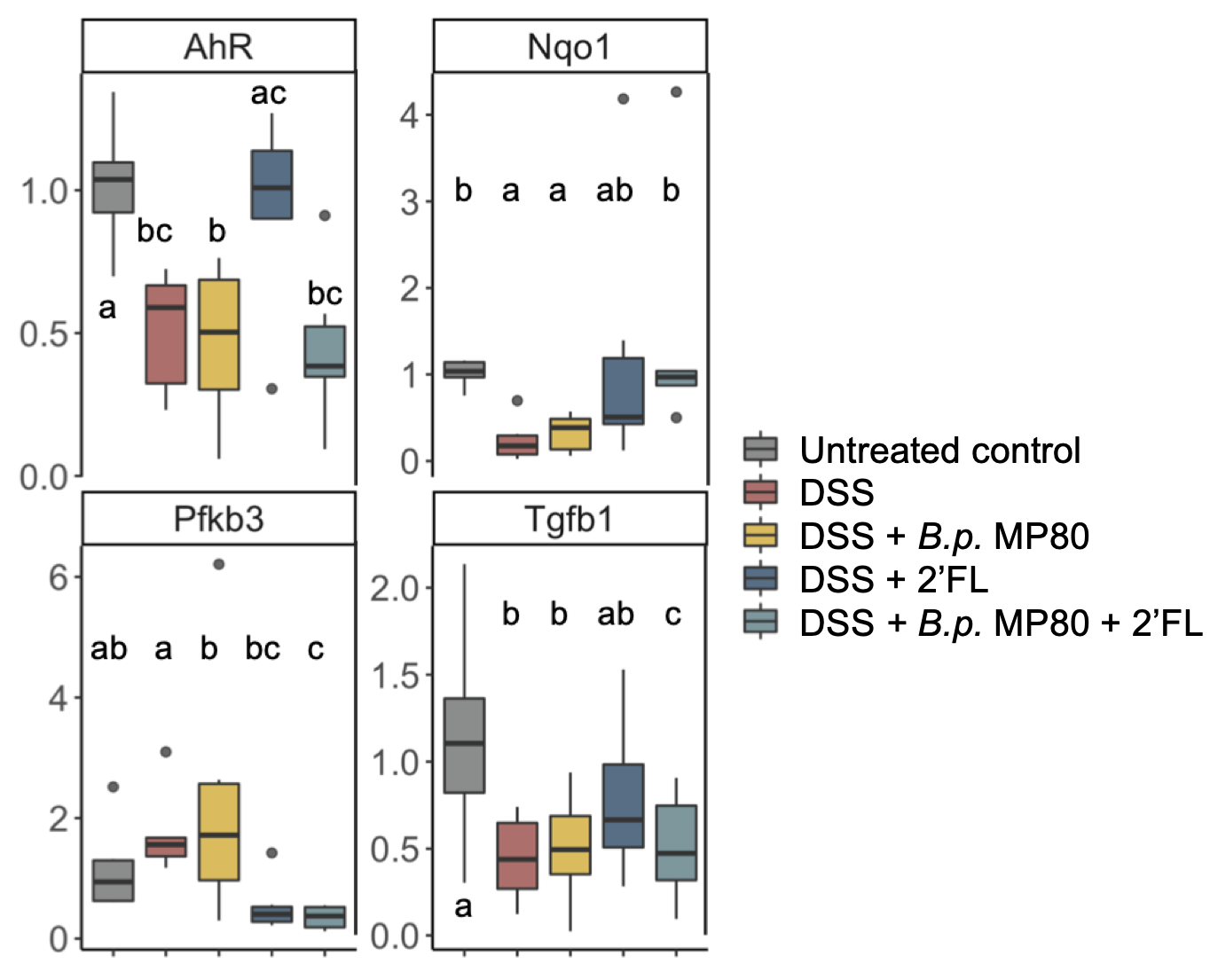

Supplement: Supplemental Material [file KGMI_A_1986666_SM4160.zip › Supplementary information/SuppFig8.tiff]

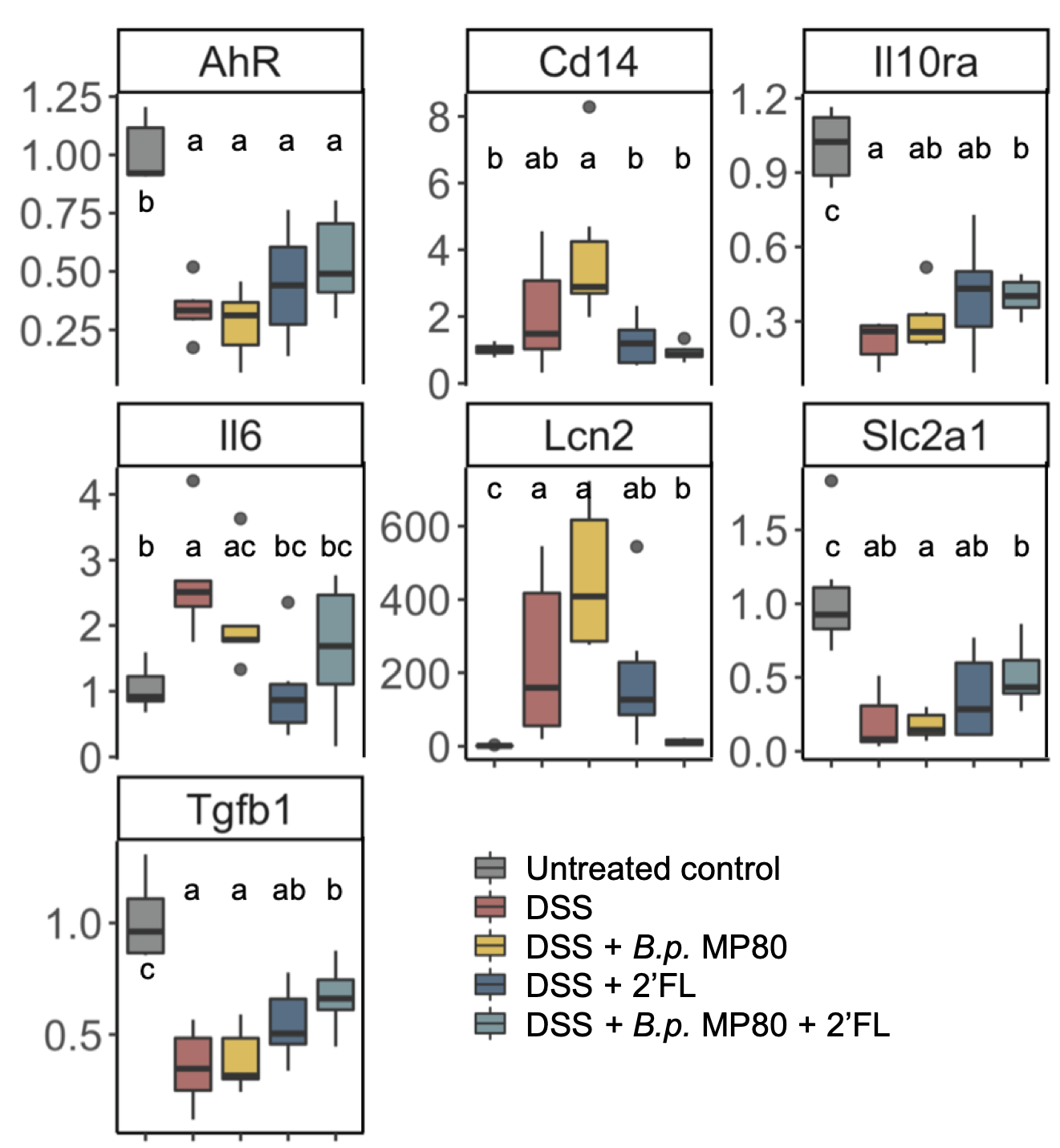

Supplement: Supplemental Material [file KGMI_A_1986666_SM4160.zip › Supplementary information/SuppFig9.tiff]
