# Supplementary material for: Bifidobacterium catabolism of human milk oligosaccharides overrides endogenous competitive exclusion driving colonization and protection
Source: Gut Microbes. 2021 Oct 27;13(1):1986666. doi: 10.1080/19490976.2021.1986666 (PMC8555557; doi:10.1080/19490976.2021.1986666)
Supplement: Supplemental Material [file KGMI_A_1986666_SM4160.zip › Supplementary information/HeissEhrlich_SupplementalFinal.docx]

**SUPPLEMENTAL METHODS**

***Study Design***

Sample size was determined based on a preliminary *Bifidobacterium* + 2’FL experiment that resulted in consistent persistence. Therefore, we conducted two *B.p.* MP80 persistence experiments at two distinct time points to account for cage effects and different indigenous mouse microbiotas; *n* = 3 mice per treatment per experiment. Two separate DSS experiments were conducted for replication with an *n* = 3 for each. The preliminary experiment also revealed that after a washout period of 10 days the supplemented *Bifidobacterium* was below the limit of detection which is how we chose our end point. Mice were randomly assigned into treatment groups 4-6 days prior to initiating experiments. Researchers served as animal caretakers and were aware of treatments provided to each cage, therefore, the mouse experiments were not conducted blind. Histological assessment was conducted blind.

***Mouse experimental design***

*Persistence experiments:* *B.p.* MP80 or *B.p.* JCM11661 were provided for 5 days by oral gavage starting from experimental day 1. 2’FL was provided in the drinking water from experimental days 1 to 10. During days 11 through 21, all treatment groups underwent a washout period with drinking water alone. Fecal samples were collected every 2-4 days throughout the experiment. For health phenotyping experiments, *B.p.* MP80 was the only *Bifidobacterium* administered.

*DSS phenotype experiments: B.p.* MP80 was provided for 4 days by oral gavage starting from experimental day 1. 2’FL was provided in the drinking water from experimental days 1 to 12. DSS was provided in the drinking water at 3% w/v during days 6-10. Fecal samples were collected every 2-4 days throughout the experiment. Food and water intakes and mouse body weights were measured daily. Mice were sacrificed on day 12.

***Preparation of bacterial inoculum and 2’-fucosyllactose for mice***

*B.p.* MP80 was previously isolated in the Mills lab from an infant fecal sample. *Bifidobacterium* strains were grown under anaerobic conditions at 37°C and propagated in de Man, Rogosa, and Sharpe media (BD Difco Microbiology, Houston, TX) supplemented with 0.05% w/v L-cysteine (Sigma-Aldrich, St. Louis, MO).  For mouse bacterial inoculation stocks, strains were separately grown, harvested at late exponential phase, washed with reduced PBS under anaerobic conditions, pelleted by centrifugation (8000 x g for 10 min), and then re-suspended in pre-reduced PBS with a final concentration of 10% v/v glycerol (pH 7). Quantitative culture revealed approximately 10^9^ CFU/ml after freeze thaw. 2’-fucosyllactose (Advanced Protein Technologies, South Korea) was diluted into sterile drinking water at a concentration of 0.10 grams per milliliter and filter sterilized.

***Quantification of bacterial strains by qPCR***

*B.p.* JCM11661 was quantified by designing strain-specific primers to target genes identified to be unique by comparing the genome against a selection of closely related strains and bacteria common to mouse gastrointestinal tracts using JGI/IMG tools (**Supplemental table 1**).^1^ Primers’ strain specificity was tested in silico by conducting a BLAST search against the NCBI database. Primers were tested using qPCR against fecal DNA from acclimation periods, and confirmed to produce no to very low background amplification. *B.p.* MP80 and *Bifidobacterium* were quantified by previously evaluated primers (**Supplemental table 1**).^2,3^ Cell numbers of strains were quantified by absolute quantification using a standard curve prepared with DNA isolated from bacterial cultures with a CFU/ml determined by quantitative culture. Standard curves were prepared by using pure cultures of each microorganism harvested at late exponential phase, determined by growth curves generated for each strain under anaerobic conditions. qPCR was performed using a 7500 Fast Real-Time PCR System (Applied Biosystems, Waltham, MA, USA), with PCR reaction volumes of 20 μl using PowerUp SYBR Green qPCR master mix (Applied Biosystems, Waltham, MA, USA), 0.8 μM primer concentrations, and 1 μl DNA template.

***^1^H-NMR metabolite sample preparation and analysis***

Colon contents were weighed, resuspended in ice cold PBS, vortexed for 2 minutes, incubated on ice for 5 min and vortexed again before centrifugation (6000 x RCF, 15 minutes, 4C). Supernatant was removed, the pellets were dried in miVac sample concentrator, and weighed to determine dry fecal weight. After an additional centrifugation step (14000xRCF, 10 minutes, 40C), the supernatants were transferred to 3 kDa filters and centrifuged again (14000xRCF, 60 minutes, 40C). 207 μL of the filtrates were transferred to a new tube, and 23 μL of an internal standard consisting of 5 mM DSS-d6 containing 0.2% NaN3 (to inhibit bacterial growth) in 99.8% D2O (for locking) was added. pH was adjusted to 6.7-6.9 with NaOH or HCl as described in He et al^4^. Sera were thawed on ice and transferred to 3 kDa filters. After 60 minutes of centrifugation (4 C, 14000x RCF) 207 μL of filtrate was combined with 23 μL of 5 mM DSS-d6. pH was adjusted to to 6.7-6.9 with NaOH or HCl before transfer to 3mm NMR tubes.

^1^H NMR spectra were acquired at 298K using the NOESY 1H presaturation experiment (‘noesypr1d’) on a Bruker Avance 600 MHz NMR spectrometer (Bruker BioSpin, Germany). Data acquisition was achieved with the following parameters; 8 dummy scans and 32 transients over a spectral width of 12 ppm and a total acquisition time of 2.5 s. Water saturation was applied during relaxation delay (2.5 s) and mixing time (100 ms). The resulting spectra were Fourier transformed with zero filling to 128 k data points and the Free Induction Decays (FIDs) were transformed with an exponential apodization function corresponding to a line broadening of 0.5 Hz. Chenomx NMR Suite v8.4 (Chenomx Inc, Edmonton, Alberta, Canada) was used to manually phase and correct baseline spectra. Each metabolite was assigned manually and quantified using Chenomx Profiler.

All statistical analyses for metabolites were completed in R (v4.0.2). Normality was assessed by generating Q-Q plots and observing deviations in the residuals in addition to Shapiro-Wilk testing. Metabolites in groups with non-normal distributions were log transformed (log(y + sqrt(y^2^ + 1))) to approximate normality. Heteroskedastic data was evaluated with Welch’s t-test, followed by Games Howell post hoc testing, whereas normally distributed data with homogeneous variances was tested with ANOVA followed by Tukey’s HSD. Effect sizes were calculated using Hedge’s g formula to account for small sample sizes with medium and large effects considered as |g|> 0.5 and > 0.8 respectively. All p-values were corrected for multiple testing by FDR correction with significance assessed as *p* < 0.05 and statistical trends considered as *p* < 0.1 (**Supplemental table 2**).

***Microbial ecosystem statistics***

Statistical analysis was performed in R (version 3.6.3).^5^ α-diversity was measured with Shannon Index values for each mouse fecal sample (vegan::diversity). Shannon values were log transformed and each mouse subject’s α-diversity geometric mean was calculated from day 8 and day 10 log values. These days were selected as they are post-*Bifidobacterium* gavage and yet during 2’FL supplementation. We used a linear regression to test the effect of *B.p.* MP80 and 2’FL on α-diversity with *B.p.* JCM11661 as the control comparison. Included in the GLM analysis (lme4::glm) were robust sandwich variance estimates (clubSandwich::vcovCR) and a degrees of freedom Satterthwaite correction (clubSandwich::coef_test).^6,7^ β-diversity was measured by Bray-Curtis distances (vegan::metaMDS, dist = “bray”) which were visualized using non-metric multidimensional scaling (NMDS) (vegan::metaMDS, k=2) with ellipses (vegan::veganCovEllipse).^8^ Differences in β-diversity were detected by checking dispersion (vegan::betadisper), a permutational multivariate ANOVA (vegan::adonis2, 999 permutations), and post hoc testing (RVAideMemoire::pairwise.perm.manova, nperm = “500”).^9^ The strata argument was used to constrain by mouse when longitudinal data was examined. Log ratio ASV differences between all treatment groups was tested with an ANOVA with Tukey’s Test (stats::aov and stats::TukeyHSD).^5^ Log ratio ASV differences between 2’FL and untreated mice was tested with a student’s t test (stats::t.test).

References

1.	Maldonado-Gómez MX, Martínez I, Bottacini F, O’Callaghan A, Ventura M, van Sinderen D, Hillmann B, Vangay P, Knights D, Hutkins RW, et al. Stable Engraftment of Bifidobacterium longum AH1206 in the Human Gut Depends on Individualized Features of the Resident Microbiome. Cell Host Microbe 2016; 20:515–26.

2.	Rinttilä T, Kassinen A, Malinen E, Krogius L, Palva A. Development of an extensive set of 16S rDNA-targeted primers for quantification of pathogenic and indigenous bacteria in faecal samples by real-time PCR. J Appl Microbiol 2004; 97:1166–77.

3.	Matsuki T, Watanabe K, Fujimoto J, Kado Y, Takada T, Matsumoto K, Tanaka R. Quantitative PCR with 16S rRNA-gene-targeted species-specific primers for analysis of human intestinal bifidobacteria. Appl Environ Microbiol 2004; 70:167–73.

4.	He X, Parenti M, Grip T, Domellöf M, Lönnerdal B, Hernell O, Timby N, Slupsky CM. Metabolic phenotype of breast-fed infants, and infants fed standard formula or bovine MFGM supplemented formula: a randomized controlled trial. Sci Rep 2019; 9:339.

5.	R Core Team. R: A Language and Environment for Statistical	Computing. R Foundation for Statistical Computing; 2020.

6.	Bates D, Mächler M, Bolker B, Walker S. Fitting linear mixed-effects models using lme4. J Stat Softw 2015; 67:1–48.

7.	Pustejovsky J. clubSandwich: Cluster-Robust (Sandwich) Variance Estimators with Small-Sample Corrections. R; 2020.

8.	Oksanen J, Blanchet FG, Friendly M, Kindt R, Legendre P, McGlinn D, Minchin PR, O’Hara RB, Simpson GL, Solymos P, et al. vegan: Community Ecology Package [Internet]. R; 2019 [cited 2020 Jul 20]. Available from: https://CRAN.R-project.org/package=vegan

9.	Hervé M. RVAideMemoire: Testing and Plotting Procedures for Biostatistics. R; 2020.

**Supplemental figure 1**. Colon content metabolites profiled by ^1^H NMR reveals differences between untreated control, 2’FL, and *B.p.* MP80 + 2’FL. Effect sizes between (A) 2’FL and untreated, (B) *B.p.* MP80 + 2’FL and untreated, and (C) *B.p.* MP80 + 2’FL and 2’FL; (D) individual metabolite concentrations for metabolites greater in *B.p.* MP80 + 2’FL treatment versus 2’FL treatment. Medium and large effect sizes (|0.5| and |0.8| respectively) are displayed as vertical lines on each plot *(n* = 6 per group)*.* The 95% confidence interval was estimated using the normal distribution. The boxplots represent medians and interquartile range (IQR) with whisker end points equal to the maximum and minimum values below or above the median at 1.5 times the IQR.

**Supplemental figure 2.** Supplemental mouse experiments to characterize *B.p.* MP80 persistence during 2’FL administration. (A) *B.p.* MP80 quantification by qPCR in an experiment varying the number of *B.p.* MP80 gavage days (*n* = 2 per treatment group); and (B) *B.p.* MP80 quantification by qPCR from a mouse experiment with varying concentrations of 2’FL provided to mice for 5 days post *B.p.* MP80 gavage (*n* = 3 per treatment group)*.* In (A) and (B), 5: final day of *B.p.* MP80 gavage; 10: final day of 2’FL supplementation; 15: washout of 2’FL, day before necropsy. The experimental groups labeled with different letters are significantly different at each time point (one-way ANOVA with Tukey’s multiple comparisons; p < 0.05).

**Supplemental figure 3**. *B.p.* MP80 microbial community effects and supplemental *Bifidobacterium* qPCR. (A) NMDS plot of the β-diversity index Bray-Curtis for *B.p.* MP80 treatment, colored by time (*n* = 6); (B) Bray Curtis matrix values from baseline (day 0) vs. final *B.p.* MP80 gavage (day 6) with a Mann Whitney test (*n* = 6 per treatment); and (C) *Bifidobacterium* quantification by qPCR in fecal DNA for 2’FL (dashed line) and *B.p.* MP80 + 2’FL (solid line) treatments with a Kruskal Wallis test (*n* = 6).

**Supplemental figure 4**. Cecum gene expression for *B.p.* MP80 persistence experiments. Untreated mice (*n* = 6-9), *B.p.* MP80 mice (*n* = 6), 2’FL mice (*n* = 6), and *B.p.* MP80 + 2’FL mice (*n* = 6). Select genes significant by Kruskal-Wallis test with FDR correction. Different letters signify statistical difference between treatments (post hoc Wilcoxon Rank Sum with FDR correction; *p* < 0.05).

**Supplemental figure 5**. Colon gene expression for *B.p.* MP80 persistence experiments. Untreated mice (*n* = 6-9), *B.p.* MP80 mice (*n* = 6), 2’FL mice (*n* = 6), and *B.p.* MP80 + 2’FL mice (*n* = 6). Select genes significant by Kruskal-Wallis test with FDR correction. Different letters signify statistical difference between treatments (post hoc Wilcoxon Rank Sum with FDR correction; *p* < 0.05).

**Supplemental figure 6**. Jejunum gene expression for *B.p.* MP80 persistence experiments. Untreated mice (*n* = 6-9), *B.p.* MP80 mice (*n* = 6), 2’FL mice (*n* = 6), and *B.p.* MP80 + 2’FL mice (*n* = 6). Select genes significant by Kruskal-Wallis test with FDR correction. Different letters signify statistical difference between treatments (post hoc Wilcoxon Rank Sum with FDR correction; *p* < 0.05).

**Supplemental figure 7**. Colon gene expression for DSS-induced colitis experiments. Untreated mice (*n* = 6), DSS mice (*n* = 6), DSS + *B.p.* MP80 mice (*n* = 6), DSS + 2’FL mice (*n* = 6), and DSS + *B.p.* MP80 + 2’FL mice (*n* = 6). Select genes significant by Kruskal-Wallis test with FDR correction. Different letters signify statistical difference between treatments (post hoc Wilcoxon Rank Sum with FDR correction; *p* < 0.05).

**Supplemental figure 8**. Jejunum gene expression from DSS-induced colitis experiments. Untreated mice (*n* = 6), DSS mice (*n* = 6), DSS + *B.p.* MP80 mice (*n* = 6), DSS + 2’FL mice (*n* = 6), and DSS + *B.p.* MP80 + 2’FL mice (*n* = 6). Select genes significant by Kruskal-Wallis test with FDR correction. Different letters signify statistical difference between treatments (post hoc Wilcoxon Rank Sum with FDR correction; *p* < 0.05).

**Supplemental figure 9**. Liver gene expression from DSS-induced colitis experiments. Untreated mice (*n* = 6), DSS mice (*n* = 6), DSS + *B.p.* MP80 mice (*n* = 6), DSS + 2’FL mice (*n* = 6), and DSS + *B.p.* MP80 + 2’FL mice (*n* = 6). Select genes significant by Kruskal-Wallis test with FDR correction. Different letters signify statistical difference between treatments (post hoc Wilcoxon Rank Sum with FDR correction; *p* < 0.05).

**Supplemental figure 10**. Heat maps of gene expression changes in DSS challenged mice in (A) liver; and (B) jejunum (*n* = 6 per treatment). Log_2_ transformed fold changes are expressed with DSS alone challenged mice values expressed relative to untreated control mice and all other DSS-challenged mice expressed relative to DSS alone challenged mice (statistical analysis contained in supplemental figures 8-9 and supplemental tables 20-23). Genes were grouped according to known function: serotonin (5-HT) regulatory targets, aryl hydrocarbon receptor (AhR) pathway targets, intestinal barrier markers, anti-inflammatory, pro-inflammatory, inflammatory modulating, and inflammatory metabolism.

**Supplemental figure 11**. Select Spearman’s correlations between colon gene expression and fecal *B.p.* MP80 abundance for treatment groups DSS + *B.p.* MP80 and DSS + *B.p.* MP80 + 2’FL at day of necropsy. Correlations and associated *p* value for inflammatory markers (A) AhR, (B) Il6, (C) Ocln, (D) Tgfb1, (E) Hrh2, (F) serum LPS binding protein, (G) Tph1, (H) NFkB, (I) colon length (*n* = 6 per treatment).

**Supplemental figure 12**. Serum metabolites assessed by ^1^H NMR reveals differences between untreated (gray), DSS (red), and DSS + *B.p.* MP80 + 2’FL (blue) (*n* = 6 per treatment). (A) Principal component analysis (PCA) of generalized log transformed data; (B) serum metabolite effect sizes between DSS + *B.p.* MP80 + 2’FL and DSS; and (C) individual metabolite concentrations by treatment group. Medium and large effect sizes (|0.5| and |0.8| respectively) are displayed as vertical lines on each plot. The 95% confidence interval was estimated using the normal distribution. The boxplots represent medians and interquartile range (IQR) with whisker end points equal to the maximum and minimum values below or above the median at 1.5 times the IQR. * signifies *p* < 0.05 after FDR correction.

**Supplemental figure 13**. Heat maps of regional brain gene expression changes in DSS challenged mice in (A) hypothalamus, (B) hippocampus, and (C) prefrontal cortex. Log_2_ transformed fold changes are expressed with DSS alone challenged mice values expressed relative to untreated control mice and all other DSS-challenged mice expressed relative to DSS alone challenged mice (*n* = 6 per treatment). Genes were grouped according to known function: neurological and pro-inflammatory.

| Primer Target  Forward and Reverse Sequences (5’ – 3’) | | Product Size (bp) | Annealing  Temp (C) | | Source | |
| --- | --- | --- | --- | --- | --- | --- |
| *Bifidobacterium*  TCGCGTC(C/T)GGTGTGAAAG  CCACATCCAGC(A/G)TCCAC | 243 | | 58 | Rinttila et al. *(53)* | |  |
| *B. catenulatum*  CGGATGCTCCGACTCCT  CGAAGGCTTGCTCCCGAT | 285 | | 58 | Matsuki et al. *(54)* | |  |
| *B .pseudocatenulatum JCM11661*  AAAACGGCGTGACCTACAAC  GGAGGTCTGGTTGGTCTTGA | 158 | | 60 | This Study | |  |
| *B. pseudocatenulatum MP80 Fucosidases*  AGTGGAATACACGTTGCGCT  CCGCATGCTGGTAGCAAAAA | 212 | | 60 | This Study | |  |

**Supplemental table 1**: List of primers used for bacterial absolute quantification (qPCR) all of which used SYBR Green ® as the detection chemistry.

|  | *p* value | | | FDR-adjusted *q* value | | | |
| --- | --- | --- | --- | --- | --- | --- | --- |
|  | Untreated – 2’FL | Untreated –  *B.p.* MP80 + 2FL | 2’FL –  *B.p.* MP80 + 2’FL | Untreated – 2’FL \| Untreated – *B.p.* MP80 + 2FL \| 2’FL – *B.p.* MP80 + 2’FL | Untreated –  *B.p.* MP80 + 2FL | 2’FL –  *B.p.* MP80 + 2’FL |  |
| 2-hydroxyisovalerate | 0.232 | **0.038** | 0.154 | 0.352 | 0.154 | 0.328 |  |
| 2-oxoglutarate | 0.140 | **0.017** | 0.215 | 0.328 | 0.145 | 0.352 |  |
| 3-methyl-2-oxo-butanoate | 0.315 | 0.146 | 0.224 | 0.426 | 0.328 | 0.352 |  |
| 5-aminopentanoate | 0.229 | **0.030** | **0.026** | 0.352 | 0.151 | 0.150 |  |
| Acetate | 0.519 | 0.664 | 0.154 | 0.622 | 0.757 | 0.328 |  |
| Alanine | 0.079 | 0.120 | **0.006** | 0.246 | 0.314 | 0.145 |  |
| Butyrate | 0.197 | 0.353 | 0.058 | 0.352 | 0.454 | 0.198 |  |
| Choline | 0.474 | 0.351 | 0.522 | 0.581 | 0.454 | 0.622 |  |
| Ethanol | 0.307 | **0.011** | **0.009** | 0.421 | 0.145 | 0.145 |  |
| Formate | **0.023** | **0.035** | 0.052 | 0.145 | 0.151 | 0.198 |  |
| Fucose | 0.144 | 0.767 | 1.000 | 0.328 | 0.839 | 1.000 |  |
| Fumarate | 0.265 | 0.964 | 0.271 | 0.376 | 0.988 | 0.378 |  |
| Glutamate | 0.174 | 0.250 | 0.059 | 0.352 | 0.362 | 0.198 |  |
| Glutamine | 0.396 | 0.198 | 0.096 | 0.494 | 0.352 | 0.277 |  |
| Isoleucine | 0.217 | **0.029** | **0.010** | 0.352 | 0.151 | 0.145 |  |
| Lactate | 0.692 | 0.846 | 0.937 | 0.770 | 0.902 | 0.973 |  |
| Leucine | 0.192 | 0.054 | **0.010** | 0.352 | 0.198 | 0.145 |  |
| Lysine | 0.130 | 0.190 | **0.021** | 0.328 | 0.352 | 0.145 |  |
| *Myo*-inositol | 0.207 | 0.904 | 0.237 | 0.352 | 0.951 | 0.352 |  |
| Propionate | 0.694 | **0.022** | **0.021** | 0.770 | 0.145 | 0.145 |  |
| 1,2-propanediol | 0.847 | 0.171 | 0.106 | 0.902 | 0.352 | 0.297 |  |
| Pyruvate | 0.190 | 0.120 | 0.997 | 0.352 | 0.314 | 1.000 |  |
| Succinate | 0.377 | 0.554 | 0.086 | 0.477 | 0.650 | 0.258 |  |
| Taurine | 0.213 | 0.334 | 0.078 | 0.352 | 0.444 | 0.246 |  |
| Tryptophan | 0.149 | 0.237 | **0.035** | 0.328 | 0.352 | 0.151 |  |
| Tyrosine | 0.239 | **0.035** | **0.011** | 0.352 | 0.151 | 0.145 |  |
| Valine | 0.607 | **0.018** | **0.013** | 0.703 | 0.145 | 0.145 |  |

**Supplemental table 2**: Colon content metabolite comparisons with Games Howell post hoc test before and after FDR adjustment for *B.p.* MP80 + 2’FL persistence experiments. Metabolites with a *p* < 0.05 are shown in bold. *q* values are FDR corrected by the number of metabolites tested (48).

|  | Beta  Coefficients | | t-statistic | | Lower  95% CI | | Upper  95% CI | | *p*-value | |
| --- | --- | --- | --- | --- | --- | --- | --- | --- | --- | --- |
| *B. p.* MP80:2’FL | -0.42 | -3.17 | | -0.73 | | -0.12 | | **0.013** | |  |
| *B.p.* MP80 | 0.14 | 6.02 | | 0.08 | | 0.21 | | **0.004** | |  |
| *B.p.* JCM11661:2’FL | -0.08 | -1.66 | | -0.22 | | 0.05 | | 0.172 | |  |

**Supplemental table 3**: Linear regression model of Shannon Index (α-diversity) values evaluating the interaction (denoted by :) between 2’FL supplementation and *B.p.* MP80 (*n* = 6) versus *B.p.* JCM11661 (*n* = 3). Robust sandwich variance estimates and degrees of Satterthwaite correction incorporated. Values in bold are *p* < 0.05.

|  | *B.p.* MP80 + 2’FL | | *B.p.* MP80 | | 2’FL | |
| --- | --- | --- | --- | --- | --- | --- |
| *B.p.* MP80 | **0.008** |  | |  | |  |
| 2’FL | 0.358 | **0.008** | |  | |  |
| Untreated | **0.008** | 0.488 | | **0.008** | |  |

**Supplemental table 4**: Pairwise comparisons of PERMANOVA for Bray Curtis values (β-diversity) evaluating differences between microbial communities of all treatments (*n* = 6 mice per treatment) on day 10, the final day of 2’FL supplementation (nperm = 200). Values in bold are *p* < 0.05.

|  | Differential | | Lower 95% CI | | Upper 95% CI | | *p value* |  |  |
| --- | --- | --- | --- | --- | --- | --- | --- | --- | --- |
| *B. p.* MP80 + 2FL – Untreated | 4.35 | 2.39 | | 6.29 | | **< 0.001** | | |  |
| *B.p.* MP80 + 2’FL – *B.p.* MP80 | 4.31 | 2.36 | | 6.26 | | **< 0.001** | | |  |
| *B.p.* MP80 + 2’FL – 2’FL | 2.34 | 0.33 | | 4.34 | | **0.017** | | |  |
| Untreated *– B. p.* MP80 | 0.04 | -2.04 | | 1.96 | | > 0.999 | | |  |
| Untreated – 2’FL | 2.01 | -4.06 | | 0.04 | | 0.056 | | |  |
| *B.p.* MP80 – 2’FL | -1.97 | -4.03 | | 0.08 | | 0.063 | | |  |

**Supplemental table 5**: Tukey’s multiple comparison testing of the log ratio of *Bifidobacteriaceae* ASVs relative to low ranked *Lachnospiraceae* and *Ruminococcaceae* ASVs by treatment. Values in bold are *p* < 0.05.

| ASV Feature ID | Bacterial Family | Log Ratio Classification |
| --- | --- | --- |
| 46ef1a1d163d392f75bd877a6b6eb7cc | *Bifidobacteriaceae* | Numerator |
| 931fff02d94342afd4b68703cebc9d13 | *Bifidobacteriaceae* | Numerator |
| ce6eabf2d84da6efcea6fbc168c92d03 | *Bifidobacteriaceae* | Numerator |
| f4f5cf8d8d238c12682888d27859fa3f | *Bifidobacteriaceae* | Numerator |
| 12fab84068d560d5ddaf5e4c1d1182fd | *Ruminococcaceae* | Denominator |
| ac6858e21fdfd6b1a2863e73180d8caf | *Lachnospiraceae* | Denominator |
| 1e22f1407ca878c2d0448f300ab7d817 | *Ruminococcaceae* | Denominator |
| 32fa1e9dfd85f3c7fe5765158d07a1fd | *Lachnospiraceae* | Denominator |
| 74804ec12dd657a69648c9da08abd597 | *Ruminococcaceae* | Denominator |
| 4e6dacec034d4b1431ef7f9e57d9490a | *Ruminococcaceae* | Denominator |
| 61dda49be7ef2e6ba371ae50a17afc10 | *Ruminococcaceae* | Denominator |
| 30584d9aed923c0c1d895d6201f19924 | *Lachnospiraceae* | Denominator |
| f6fee0996809fffc3b3b8d32845466e7 | *Lachnospiraceae* | Denominator |
| 5bcabac8d78720c0db64155569782725 | *Lachnospiraceae* | Denominator |
| f3cb623fafc2eae95b22949eebc0ac80 | *Lachnospiraceae* | Denominator |
| e72e247bda1d9d5a1e30dc21fa920ec4 | *Ruminococcaceae* | Denominator |
| 74778997f2b404d99413cdbb92be937e | *Ruminococcaceae* | Denominator |
| df2d1dcce96a59a12330e5f955a2847f | *Lachnospiraceae* | Denominator |
| 2097c2310c94b63b7aa3f1d282720fe1 | *Ruminococcaceae* | Denominator |
| 1c728cf6c1d5828eeaf57e83c39f0600 | *Ruminococcaceae* | Denominator |
| 7f9ac6505ff478702b26a9e0ca12f2e4 | *Lachnospiraceae* | Denominator |
| 9e4b28d37cdaf716c79a07f62d856852 | *Ruminococcaceae* | Denominator |
| 9873f6eaf55845b58e027d51407ee056 | *Lachnospiraceae* | Denominator |
| 39a2666452fe7cb00aa9d17a2ba9b7d8 | *Lachnospiraceae* | Denominator |
| 584f07ed2c373c887dc223e744047d5c | *Ruminococcaceae* | Denominator |
| ae439e26c93eacd8002fd7bc06eafe22 | *Lachnospiraceae* | Denominator |
| 501737a85ba32f8232caf28f6ff3b84e | *Ruminococcaceae* | Denominator |
| e29666e119130822c19d1415430c039b | *Lachnospiraceae* | Denominator |
| 4d0d4fcf2265051d832c34fea4396739 | *Lachnospiraceae* | Denominator |
| 6d15b963bd8adbf52bb4d46bd85e6f59 | *Lachnospiraceae* | Denominator |
| c2a737924e989ece22452a86bbeaf775 | *Lachnospiraceae* | Denominator |
| 6c9202c2a41df232d8a39d13017c4982 | *Lachnospiraceae* | Denominator |
| acb734be7481d4f49f61bb4f3d46b92c | *Lachnospiraceae* | Denominator |
| 6df4225cb7b6423e58e4a82daff43001 | *Ruminococcaceae* | Denominator |
| 438545090161435c29c5c96c5181a746 | *Ruminococcaceae* | Denominator |
| ec0ede08845dad4df7113237e1838fe5 | *Ruminococcaceae* | Denominator |
| 5872de8f236ebe7df78bdcb54cb6837e | *Lachnospiraceae* | Denominator |
| cf15f944ae602f0e3111e32570d0b545 | *Lachnospiraceae* | Denominator |
| bef4daf8c571b34fee9f7e9219d4e584 | *Lachnospiraceae* | Denominator |
| dd6593e0cd2e878bf2220f5d8670f8ca | *Lachnospiraceae* | Denominator |
| 556fefa5d5dc7afdeb72b5bebade25a0 | *Lachnospiraceae* | Denominator |
| a6788561949b316d9018742a8d14b638 | *Lachnospiraceae* | Denominator |
| 0bb16df5759b121505f224e3efd23072 | *Lachnospiraceae* | Denominator |
| 28018a6f7c334b8fb92d616bac5cbdcb | *Lachnospiraceae* | Denominator |
| 0e098e232ddc310d4b654d82d1b0c82e | *Lachnospiraceae* | Denominator |
| 127a8e50dfc16470d189ed4173e4badb | *Lachnospiraceae* | Denominator |
| 32495f2b8edbb6a142b3f8a6a081857a | *Lachnospiraceae* | Denominator |
| 640f30c84bf18f225e048bed207e63c7 | *Lachnospiraceae* | Denominator |
| 6022dc18fc918874fe68e467d878af20 | *Lachnospiraceae* | Denominator |
| db74e3bce11f81adf607afa3ade2cc70 | *Ruminococcaceae* | Denominator |
| 68b76711c0d2a0cd069e729df926548f | *Lachnospiraceae* | Denominator |
| ed92e36ec94dc479c844e4df424908ce | *Ruminococcaceae* | Denominator |

**Supplemental table 6**: Feature IDs selected using Songbird to generate a log ratio to calculate differential abundance between *B.p.* MP80 + 2’FL treatment and treatments *B.p.* MP80, 2’FL, and untreated control. *Lachnospiraceae* and *Ruminococcaceae* ASVs were selected from the lowest 25% ranked ASVs.

| ASV Feature ID | Bacterial Family | Log Ratio Classification |
| --- | --- | --- |
| 46ef1a1d163d392f75bd877a6b6eb7cc | *Bifidobacteriaceae* | Numerator |
| 59044bba9492389101d12888fb981b19 | *Bacteroidaceae* | Numerator |
| 61143439625764e98d254209f9d10304 | *Bacteroidaceae* | Numerator |
| ce6eabf2d84da6efcea6fbc168c92d03 | *Bifidobacteriaceae* | Numerator |
| ae439e26c93eacd8002fd7bc06eafe22 | *Lachnospiraceae* | Denominator |
| b73a98840df0e8eabd9e04684a9fd40e | *Lachnospiraceae* | Denominator |
| ac6858e21fdfd6b1a2863e73180d8caf | *Lachnospiraceae* | Denominator |
| 1c728cf6c1d5828eeaf57e83c39f0600 | *Ruminococcaceae* | Denominator |
| 34d57454243e07e31b65c276f869b6a6 | *Lachnospiraceae* | Denominator |
| 12fab84068d560d5ddaf5e4c1d1182fd | *Ruminococcaceae* | Denominator |
| 2a2b8eb747cafad379a264737faeca63 | *Lachnospiraceae* | Denominator |
| f6fee0996809fffc3b3b8d32845466e7 | *Lachnospiraceae* | Denominator |
| 1e22f1407ca878c2d0448f300ab7d817 | *Ruminococcaceae* | Denominator |
| 1dd2c20574f2b4bf58db53648b30cd0c | *Lachnospiraceae* | Denominator |
| 9e4b28d37cdaf716c79a07f62d856852 | *Ruminococcaceae* | Denominator |
| 356afb7a82f0050f844d2e6d907c3fa2 | *Lachnospiraceae* | Denominator |
| 6d15b963bd8adbf52bb4d46bd85e6f59 | *Lachnospiraceae* | Denominator |
| 35cb1ff0165c6176cf381d53d0652d72 | *Lachnospiraceae* | Denominator |
| e72e247bda1d9d5a1e30dc21fa920ec4 | *Ruminococcaceae* | Denominator |
| 9873f6eaf55845b58e027d51407ee056 | *Lachnospiraceae* | Denominator |
| 28018a6f7c334b8fb92d616bac5cbdcb | *Lachnospiraceae* | Denominator |
| f3cb623fafc2eae95b22949eebc0ac80 | *Lachnospiraceae* | Denominator |
| dd6593e0cd2e878bf2220f5d8670f8ca | *Lachnospiraceae* | Denominator |
| 88cd10c71ba0e12387ef9c6e5a3c602b | *Ruminococcaceae* | Denominator |
| d7ec692f6ed54afb0111f6ad30945350 | *Lachnospiraceae* | Denominator |
| d8537b5c60fa537157d25264c3734bdf | *Ruminococcaceae* | Denominator |
| a6788561949b316d9018742a8d14b638 | *Lachnospiraceae* | Denominator |
| 5872de8f236ebe7df78bdcb54cb6837e | *Lachnospiraceae* | Denominator |
| 718230c252b3e24f132152ee42b6b3f0 | *Lachnospiraceae* | Denominator |
| 74804ec12dd657a69648c9da08abd597 | *Ruminococcaceae* | Denominator |
| ed92e36ec94dc479c844e4df424908ce | *Ruminococcaceae* | Denominator |
| 95bef9cd263b65a4710cf63b52347d9d | *Ruminococcaceae* | Denominator |
| be5338ba42939fb0be20c865f881ae42 | *Lachnospiraceae* | Denominator |
| b4043718f2da6bf61bbeaabdef4c9ff9 | *Lachnospiraceae* | Denominator |
| 5bcabac8d78720c0db64155569782725 | *Lachnospiraceae* | Denominator |
| aa935103e3f612d4a45a649b5acab4e0 | *Ruminococcaceae* | Denominator |
| cf15f944ae602f0e3111e32570d0b545 | *Lachnospiraceae* | Denominator |
| e760e34a170a081eeeb51eda08ba47c3 | *Ruminococcaceae* | Denominator |
| 2ab756065c27e6163ad4259a5609c5b1 | *Lachnospiraceae* | Denominator |
| c2a737924e989ece22452a86bbeaf775 | *Lachnospiraceae* | Denominator |
| ec0ede08845dad4df7113237e1838fe5 | *Ruminococcaceae* | Denominator |
| 48b1bd255e8dec337da1d05e1bc27780 | *Lachnospiraceae* | Denominator |
| 5f5944bbc4632122fcd45e2a8ab335b9 | *Lachnospiraceae* | Denominator |

**Supplemental table 7**: Feature IDs selected using Songbird to generate a log ratio to calculate differential abundance between 2’FL and untreated control mice. *Lachnospiraceae* and *Ruminococcaceae* ASVs were selected from the lowest 25% ranked ASVs.

| *Code* | *Genes* | *Forward (5’ – 3’)* | *Reverse (5’ – 3’)* |
| --- | --- | --- | --- |
| Ahr | aryl hydrocarbon receptor | CTGACAGAAATGGAGGCCAGGA | AGAAGACCAAGGCATCTGCTGT |
| Bdef2 | beta-defensin 2 | CACTCCAGCTGTTGGAAGTTTA | GCTCTGACACAGTACCCTCC |
| Ccl2 | hemokine (C-C motif) ligand 2 | CCACTCACCTGCTGCTACTCAT | GAAGTGCTTGAGGTGGTTGTGG |
| Cd14 | [cluster of differentiation 14](https://en.wikipedia.org/wiki/Cluster_of_differentiation) | ACAACAGGCTGGATAGGAACCC | TTGACGAGGACCCTCAGAAACC |
| Cyp1a1 | Cytochrome P450, family 1, subfamily A | ATTCCTGTCCTCCGTTACCTGC | ATGAGGCTGTCTGTGATGTCCC |
| Foxp3 | forkhead box P3 | AGGCTGATCCCCCTCTAGCA | AGCAGAGCTGTGTCTGGTGG |
| Gdnf | Glial cell-derived neurotrophic factor | AGAGGGGCAAAAATCGGGG | CCGCTGCAATATCGAAAGATCA |
| Gfap | Glial fibrillary acidic protein | GCGGCTGCTCTTGTAGTGAA | AGGGCCACATACCAGTAAAAC |
| Gpx2 | Glutathione peroxidase 2 | TCGGACATCAGGAGAACTGTC | TGCCCATTGACGTCACACTT |
| Hmox1 | heme oxygenase 1 | GCCCCACCAAGTTCAAACAG | GCTCCTCAAACAGCTCAATGT |
| H2R | Histamine Receptor H2 | TGCCCTTCTCTGCCATTTACCA | TTGAGGATGGAAGCTGTGCAGA |
| Iba1 | Ionized calcium binding adaptor molecule 1 | CGCTCCTAGTGGGTCACATC | ATCAGCCATAGCAACCCCAT |
| Il-10 | Interleukin 10 | TTCCCTGGGTGAGAAGCTGAAG | TCACTCTTCACCTGCTCCACTG |
| Il-10r | Interleukin 10 receptor | CATTGCATACGGGACAGAACTGC | TTTCCGTACTGTTTGAGGGCCA |
| Il-22 | Interleukin 22 | ACTTGTGCGATCTCTGATGGCT | TATGGCTGCTGGAAGTTGGACA |
| Il1b | Interleukin 1 beta | TGCCACCTTTTGACAGTGATGA | CCCAGGTCAAAGGTTTGGAAGC |
| Il6 | Interleukin 6 | ACCCCAATTTCCAATGCTCTCC | AACGCACTAGGTTTGCCGAGTA |
| Myd88 | Myeloid differentiation primary response 88 | CCTGAGCAACTAGGACTGCCTT | GGAGGCATGTGTGTACTGAGGT |
| Ngf1 | nerve growth factor 1 | GGAGCGCATCGAGTTTTGG | CCTCACTGCGGCCAGTATAG |
| Nqo1 | NAD(P)H dehydrogenase [quinone] 1 | GGTAGCGGCTCCATGTACTC | CGCAGGATGCCACTCTGAAT |
| Ocln | Occludin | ATGGCAAGCGATCATACCCAGA | GGTTACCAATTGCTGCTGTACCG |
| Pfkfb3 | 6-Phosphofructo-2-Kinase/Fructose-2,6-Biphosphatase 3 | CCCAGAGCCGGGTACAGAA | GGGGAGTTGGTCAGCTTCG |
| Rpl13a | 60S ribosomal protein L13a | CTGCTGCTCTCAAGGTTGTTCG | TGTCACTGCCTGGTACTTCCAC |
| Rplp0 | Ribosomal Protein Lateral Stalk Subunit P0 | CCATGATGCGCAAGGCTATCAG | TGCCTCTGGAGATTTTCGTGGT |
| Slc2a1 | facilitated glucose transporter member 1 | CAGTTCGGCTATAACACTGGTG | GCCCCCGACAGAGAAGATG |
| Slc64a | sodium-dependent serotonin transporter | TTGATGAGTCCACCCCAACTCC | AATGCGCTCCTTAAGTGTCCCT |
| Tgfb | Transforming growth factor beta | AGGAGACGGAATACAGGGCT | GGGGCTGATCCCGTTGATTT |
| Tjp | tight junction protein | AGCCTTGAACTTTGACCTCTGC | ACAGAAATCGTGCTGATGTGCC |
| Tnf-a | tumor necrosis factor alpha | TTCGAGTGACAAGCCTGTAGCC | GATAGCAAATCGGCTGACGGTG |
| Tph1 | [tryptophan hydroxylase 1](https://en.wikipedia.org/wiki/Tryptophan_hydroxylase) | CTACACTCCAGAGCCAGACACC | GTCTCCTCTGAAGCTCCAAGGG |

**Supplemental table 8**: Primers and full gene name for qRT-PCR of mouse tissue samples.

|  | Untreated | | | *B.p.* MP80 | | | | 2’FL | | | | *B.p.* MP80 + 2’FL | | | |  |
| --- | --- | --- | --- | --- | --- | --- | --- | --- | --- | --- | --- | --- | --- | --- | --- | --- |
| Starting Weight (g) | 24.7 | ± | 1.05 ^a^ | | 21.2 | ± | 0.54 ^b^ | | 22.4 | ± | 0.68 ^ab^ | | 24.5 | ± | 0.67 ^a^ | |
| Ending Weight (g) | 27.4 | ± | 1.20 ^a^ | | 22.9 | ± | 0.63 ^b^ | | 23.9 | ± | 0.67 ^bc^ | | 27.2 | ± | 0.90 ^ac^ | |
| Body Weight Change (%) | 9.9 | ± | 1.83 ^a^ | | 7.3 | ± | 0.74 ^a^ | | 6.4 | ± | 0.53 ^a^ | | 9.7 | ± | 1.79 ^a^ | |
| Spleen Weight (mg/g) | 3.3 | ± | 0.26 ^a^ | | 3.5 | ± | 0.22 ^a^ | | 3.1 | ± | 0.07 ^a^ | | 3.0 | ± | 0.15 ^a^ | |
| Liver Weight (mg/g) | 54.4 | ± | 3.55 ^a^ | | 51.3 | ± | 3.00 ^a^ | | 52.4 | ± | 1.51 ^a^ | | 51.4 | ± | 0.75 ^a^ | |
| Cecum Weight (w/ content) (mg/g) | 17.0 | ± | 0.86 ^a^ | | 16.9 | ± | 2.12 ^a^ | | 26.8 | ± | 2.26 ^a^ | | 47.6 | ± | 5.16 ^b^ | |
| Cecum Weight (w/o content) (mg/g) | 5.0 | ± | 0.32 ^a^ | | 4.7 | ± | 0.28 ^a^ | | 8.1 | ± | 0.74 ^b^ | | 9.8 | ± | 0.75 ^b^ | |
| LPS Binding Protein (unit) | 9.3 | ± | 1.35 ^a^ | | 4.1 | ± | 0.37 ^b^ | | 6.5 | ± | 0.43 ^ab^ | | 8.6 | ± | 0.89 ^a^ | |
| *Paracellular Perm. Ileum (ng/cm^2^/hr) | 2664 | ± | 503 ^a^ | | 3503 | ± | 1844 ^a^ | | 3573 | ± | 744 ^a^ | | 3147 | ± | 1121 ^a^ | |
| Transcellular Perm. Ileum (ng/cm^2^/hr) | 1357 | ± | 337 ^a^ | | 1120 | ± | 346 ^a^ | | 1641 | ± | 671 ^a^ | | 1225 | ± | 528 ^a^ | |
| Paracellular Perm. Colon (ng/cm^2^/hr) | 2143 | ± | 638 ^a^ | | 1988 | ± | 125 ^a^ | | 1204 | ± | 194 ^a^ | | 1527 | ± | 169 ^a^ | |
| Transcellular Perm. Colon (ng/cm^2^/hr) | 1288 | ± | 311 ^a^ | | 1281 | ± | 208 ^a^ | | 376 | ± | 77.4 ^b^ | | 668 | ± | 144 ^ab^ | |
| Food Intake (g/day/cage) | 9.83 | ± | 0.34 ^a^ | | 9.47 | ± | 0.57 ^a^ | | 11.3 | ± | 2.79 ^a^ | | 9.3 | ± | 1.04 ^a^ | |
| Water Intake (ml/day/mouse) | 6.36 | ± | 0.25 ^a^ | | 5.02 | ± | 0.66 ^a^ | | 5.08 | ± | 0.32 ^a^ | | 5.75 | ± | 0.29 ^a^ | |

*Permeability measures conducted using ussing chambers.

**Supplemental table 9**: Healthy mouse physiological data expressed as means +/- SEM and analyzed by one-way ANOVA with Tukey’s multiple comparisons.

|  |  | Untreated | | |  | *B.p* MP80 | | |  | 2'FL | | |  | *B.p* MP80 + 2'FL | | |
| --- | --- | --- | --- | --- | --- | --- | --- | --- | --- | --- | --- | --- | --- | --- | --- | --- |
| Ahr |  | 1.11 | ± | 0.13 |  | 0.81 | ± | 0.03 |  | 0.87 | ± | 0.27 |  | 1.20 | ± | 0.28 |
| **Bdef2** |  | 0.97 | ± | 0.23 |  | 1.00 | ± | 0.35 |  | 3.82 | ± | 1.05 |  | 2.31 | ± | 0.27 |
| Ccl2 |  | 0.93 | ± | 0.11 |  | 1.13 | ± | 0.17 |  | 0.70 | ± | 0.16 |  | 1.46 | ± | 0.22 |
| Cd14 |  | 1.13 | ± | 0.11 |  | 1.26 | ± | 0.05 |  | 0.96 | ± | 0.18 |  | 1.13 | ± | 0.11 |
| Cyp1a1 |  | 1.51 | ± | 0.51 |  | 2.57 | ± | 0.51 |  | 1.65 | ± | 0.43 |  | 2.27 | ± | 0.59 |
| Fox3p |  | 1.01 | ± | 0.06 |  | 1.14 | ± | 0.14 |  | 1.19 | ± | 0.21 |  | 1.23 | ± | 0.14 |
| Gpx2 |  | 1.03 | ± | 0.11 |  | 0.87 | ± | 0.04 |  | 1.32 | ± | 0.54 |  | 1.00 | ± | 0.10 |
| Hmox1 |  | 1.23 | ± | 0.22 |  | 0.97 | ± | 0.22 |  | 0.87 | ± | 0.09 |  | 1.03 | ± | 0.17 |
| Hrh2 |  | 1.02 | ± | 0.21 |  | 0.65 | ± | 0.07 |  | 1.09 | ± | 0.15 |  | 1.57 | ± | 0.20 |
| Il10 |  | 0.99 | ± | 0.11 |  | 0.92 | ± | 0.17 |  | 0.40 | ± | 0.12 |  | 0.66 | ± | 0.08 |
| Il10ra |  | 1.05 | ± | 0.15 |  | 0.40 | ± | 0.12 |  | 0.92 | ± | 0.17 |  | 0.66 | ± | 0.08 |
| **Il1b** |  | 1.04 | ± | 0.12 |  | 1.54 | ± | 0.18 |  | 0.87 | ± | 0.18 |  | 0.51 | ± | 0.07 |
| Il22 |  | 1.15 | ± | 0.32 |  | 1.05 | ± | 0.21 |  | 1.48 | ± | 0.23 |  | 2.00 | ± | 0.26 |
| Il6 |  | 1.07 | ± | 0.19 |  | 1.06 | ± | 0.16 |  | 1.34 | ± | 0.54 |  | 1.32 | ± | 0.12 |
| Lcn2 |  | 1.08 | ± | 0.17 |  | 0.81 | ± | 0.09 |  | 0.91 | ± | 0.27 |  | 1.27 | ± | 0.25 |
| Myd88 |  | 1.10 | ± | 0.16 |  | 0.92 | ± | 0.07 |  | 0.84 | ± | 0.19 |  | 1.07 | ± | 0.16 |
| Nqo1 |  | 1.07 | ± | 0.17 |  | 1.22 | ± | 0.09 |  | 1.13 | ± | 0.18 |  | 1.05 | ± | 0.11 |
| Ocln |  | 1.01 | ± | 0.12 |  | 0.86 | ± | 0.05 |  | 1.08 | ± | 0.34 |  | 1.01 | ± | 0.14 |
| Pfkb3 |  | 1.12 | ± | 0.11 |  | 0.92 | ± | 0.06 |  | 0.78 | ± | 0.12 |  | 0.61 | ± | 0.08 |
| Sl6a4 |  | 1.13 | ± | 0.33 |  | 0.76 | ± | 0.18 |  | 1.44 | ± | 0.32 |  | 1.16 | ± | 0.36 |
| Slc2a1 |  | 1.07 | ± | 0.18 |  | 0.81 | ± | 0.11 |  | 0.82 | ± | 0.21 |  | 0.66 | ± | 0.15 |
| Tgfb |  | 1.04 | ± | 0.10 |  | 0.78 | ± | 0.10 |  | 1.02 | ± | 0.21 |  | 1.01 | ± | 0.20 |
| Tjp1 |  | 0.95 | ± | 0.07 |  | 0.81 | ± | 0.07 |  | 1.02 | ± | 0.26 |  | 1.02 | ± | 0.08 |
| Tnfa |  | 1.30 | ± | 0.31 |  | 1.11 | ± | 0.13 |  | 1.06 | ± | 0.38 |  | 0.86 | ± | 0.18 |
| Tph1 |  | 1.04 | ± | 0.11 |  | 1.39 | ± | 0.15 |  | 1.07 | ± | 0.40 |  | 0.83 | ± | 0.13 |

**Supplemental table 10**: Colon tissue gene expression data expressed as means +/- SEM and analyzed by Kruskal-Wallis test with FDR corrected *p* values (genes with *p* values < 0.05 in bold).

|  | Untreated – | Untreated – | Untreated – | *B.p.* MP80 – | *B.p.* MP80 – | 2’FL – |
| --- | --- | --- | --- | --- | --- | --- |
|  | *B.p.* MP80 | 2’FL | *B.p.* MP80 | 2’FL | *B.p.* MP80 | *B.p.* MP80 |
|  |  |  | 2'FL |  | 2’FL | 2’FL |
| Bdef2 | **0.009** | 0.940 | **0.026** | **0.036** | 0.370 | 0.052 |
| Il1b | 0.420 | 0.062 | **0.009** | 0.062 | 0.180 | **0.009** |

**Supplemental table 11**: Colon tissue gene expression *p* values calculated by post hoc Wilcoxon Rank Sum test with FDR corrected *p* values. Genes selected based on Kruskal Wallis test with FDR correction and bold values are *p* < 0.05.

|  |  | Untreated | | |  | *B.p.* MP80 | | |  | 2'FL | | |  | *B.p* MP80 + 2'FL | | |
| --- | --- | --- | --- | --- | --- | --- | --- | --- | --- | --- | --- | --- | --- | --- | --- | --- |
| AhR |  | 1.05 | ± | 0.13 |  | 1.02 | ± | 0.18 |  | 1.95 | ± | 0.33 |  | 1.69 | ± | 0.13 |
| Ccl2 |  | 0.83 | ± | 0.14 |  | 1.04 | ± | 0.14 |  | 0.89 | ± | 0.18 |  | 1.19 | ± | 0.35 |
| Cd14 |  | 0.98 | ± | 0.11 |  | 0.95 | ± | 0.12 |  | 0.75 | ± | 0.15 |  | 0.69 | ± | 0.08 |
| Cyp1a1 |  | 1.09 | ± | 0.35 |  | 1.05 | ± | 0.24 |  | 1.32 | ± | 0.68 |  | 0.32 | ± | 0.05 |
| Foxp3 |  | 1.04 | ± | 0.11 |  | 0.82 | ± | 0.11 |  | 0.84 | ± | 0.13 |  | 0.58 | ± | 0.08 |
| Gpx2 |  | 1.03 | ± | 0.10 |  | 0.66 | ± | 0.11 |  | 1.30 | ± | 0.18 |  | 1.09 | ± | 0.12 |
| Hmox1 |  | 1.03 | ± | 0.09 |  | 0.79 | ± | 0.17 |  | 1.36 | ± | 0.16 |  | 1.09 | ± | 0.12 |
| Hrh2 |  | 1.14 | ± | 0.16 |  | 1.70 | ± | 0.18 |  | 1.19 | ± | 0.23 |  | 0.83 | ± | 0.08 |
| Il10 |  | 0.88 | ± | 0.08 |  | 1.31 | ± | 0.15 |  | 1.29 | ± | 0.38 |  | 1.34 | ± | 0.26 |
| Il10ra |  | 1.11 | ± | 0.17 |  | 0.99 | ± | 0.10 |  | 1.40 | ± | 0.31 |  | 1.65 | ± | 0.17 |
| Il1b |  | 1.04 | ± | 0.10 |  | 0.85 | ± | 0.14 |  | 1.55 | ± | 0.31 |  | 1.63 | ± | 0.36 |
| Il22 |  | 0.93 | ± | 0.10 |  | 1.78 | ± | 0.82 |  | 0.93 | ± | 0.15 |  | 0.54 | ± | 0.21 |
| Il6 |  | 1.00 | ± | 0.14 |  | 1.44 | ± | 0.32 |  | 0.75 | ± | 0.17 |  | 0.49 | ± | 0.09 |
| Lcn2 |  | 0.93 | ± | 0.12 |  | 1.04 | ± | 0.26 |  | 1.41 | ± | 0.26 |  | 1.84 | ± | 0.80 |
| Myd88 |  | 1.09 | ± | 0.17 |  | 1.25 | ± | 0.16 |  | 1.36 | ± | 0.19 |  | 1.40 | ± | 0.09 |
| Nqo1 |  | 1.01 | ± | 0.06 |  | 0.86 | ± | 0.07 |  | 1.22 | ± | 0.10 |  | 1.10 | ± | 0.11 |
| Ocln |  | 1.00 | ± | 0.13 |  | 1.20 | ± | 0.16 |  | 1.05 | ± | 0.13 |  | 1.32 | ± | 0.08 |
| **Pfkb3** |  | 1.11 | ± | 0.21 |  | 0.16 | ± | 0.02 |  | 1.87 | ± | 0.43 |  | 1.77 | ± | 0.72 |
| **Slc2a1** |  | 1.01 | ± | 0.06 |  | 0.28 | ± | 0.02 |  | 1.20 | ± | 0.16 |  | 1.31 | ± | 0.03 |
| **Slc6a4** |  | 1.10 | ± | 0.09 |  | 1.27 | ± | 0.21 |  | 2.37 | ± | 0.34 |  | 2.44 | ± | 0.38 |
| Tgfb |  | 1.07 | ± | 0.14 |  | 0.76 | ± | 0.12 |  | 1.29 | ± | 0.19 |  | 0.94 | ± | 0.04 |
| Tjp1 |  | 1.09 | ± | 0.11 |  | 1.18 | ± | 0.09 |  | 1.00 | ± | 0.24 |  | 0.74 | ± | 0.04 |
| Tnf |  | 0.98 | ± | 0.31 |  | 0.90 | ± | 0.25 |  | 5.33 | ± | 2.30 |  | 1.14 | ± | 0.37 |
| Tph1 |  | 1.33 | ± | 0.13 |  | 1.49 | ± | 0.22 |  | 1.18 | ± | 0.43 |  | 1.38 | ± | 0.32 |

**Supplemental table 12**: Jejunum tissue gene expression data expressed as means +/- SEM and analyzed by Kruskal-Wallis test with FDR corrected *p* values (genes with *p* values < 0.05 in bold).

|  | Untreated – | Untreated – | Untreated – | *B.p.* MP80 – | *B.p.* MP80 – | 2’FL – |
| --- | --- | --- | --- | --- | --- | --- |
|  | *B.p.* MP80 | 2’FL | *B.p.* MP80 | 2’FL | *B.p.* MP80 | *B.p.* MP80 |
|  |  |  | 2'FL |  | 2’FL | 2’FL |
| Pfkb3 | **0.004** | 0.340 | 0.850 | **0.005** | **0.005** | 0.870 |
| Slc2a1 | **0.004** | 0.590 | **0.006** | **0.005** | **0.005** | 0.710 |
| Slc6a4 | 0.450 | 0.005 | **0.004** | **0.043** | **0.016** | 0.940 |

**Supplemental table 13**: Jejunum tissue gene expression *p* values calculated by post hoc Wilcoxon Rank Sum test with FDR corrected *p* values. Genes selected based on Kruskal Wallis test with FDR correction and bold values are *p* < 0.05.

|  |  | Untreated | | |  | *B.p* MP80 | | |  | 2'FL | | |  | *B.p* MP80 + 2'FL | | |
| --- | --- | --- | --- | --- | --- | --- | --- | --- | --- | --- | --- | --- | --- | --- | --- | --- |
| AhR |  | 0.94 | ± | 0.06 |  | 0.83 | ± | 0.07 |  | 0.96 | ± | 0.14 |  | 0.960 | ± | 0.150 |
| Ccl2 |  | 1.10 | ± | 0.15 |  | 1.40 | ± | 0.34 |  | 1.01 | ± | 0.18 |  | 1.490 | ± | 0.270 |
| **Cd14** |  | 1.01 | ± | 0.07 |  | 0.78 | ± | 0.04 |  | 0.90 | ± | 0.11 |  | 1.420 | ± | 0.120 |
| **Cyp1a1** |  | 1.42 | ± | 0.65 |  | 0.98 | ± | 0.08 |  | 0.72 | ± | 0.25 |  | 0.270 | ± | 0.070 |
| Fox3p |  | 1.05 | ± | 0.12 |  | 1.33 | ± | 0.23 |  | 1.33 | ± | 0.13 |  | 1.300 | ± | 0.170 |
| **Gpx2** |  | 1.16 | ± | 0.21 |  | 1.15 | ± | 0.28 |  | 3.46 | ± | 0.82 |  | 3.950 | ± | 0.480 |
| **Hmox1** |  | 1.08 | ± | 0.14 |  | 2.30 | ± | 0.80 |  | 2.84 | ± | 0.47 |  | 2.680 | ± | 0.320 |
| Hrh2 |  | 0.93 | ± | 0.13 |  | 1.21 | ± | 0.24 |  | 0.85 | ± | 0.19 |  | 0.900 | ± | 0.200 |
| **Il10** |  | 1.10 | ± | 0.15 |  | 1.94 | ± | 0.30 |  | 2.19 | ± | 0.35 |  | 2.030 | ± | 0.350 |
| **Il10ra** |  | 1.06 | ± | 0.12 |  | 1.83 | ± | 0.28 |  | 1.73 | ± | 0.27 |  | 2.180 | ± | 0.250 |
| Il1b |  | 1.00 | ± | 0.11 |  | 1.77 | ± | 0.60 |  | 0.95 | ± | 0.32 |  | 2.370 | ± | 0.860 |
| Il22 |  | 0.95 | ± | 0.10 |  | 1.41 | ± | 0.54 |  | 0.83 | ± | 0.18 |  | 1.270 | ± | 0.410 |
| **Il6** |  | 0.98 | ± | 0.07 |  | 3.05 | ± | 1.10 |  | 1.36 | ± | 0.22 |  | 1.840 | ± | 0.310 |
| **Myd88** |  | 1.14 | ± | 0.19 |  | 0.73 | ± | 0.16 |  | 4.38 | ± | 0.73 |  | 3.870 | ± | 0.800 |
| **Nqo1** |  | 1.08 | ± | 0.13 |  | 1.17 | ± | 0.12 |  | 2.18 | ± | 0.37 |  | 2.540 | ± | 0.370 |
| **Pfkb3** |  | 1.11 | ± | 0.21 |  | 1.07 | ± | 0.20 |  | 3.08 | ± | 0.40 |  | 3.580 | ± | 0.910 |
| **Slc2a1** |  | 1.07 | ± | 0.15 |  | 0.80 | ± | 0.09 |  | 3.32 | ± | 0.47 |  | 3.560 | ± | 0.390 |
| Tnfa |  | 1.03 | ± | 0.13 |  | 1.07 | ± | 0.17 |  | 0.88 | ± | 0.16 |  | 0.920 | ± | 0.130 |

**Supplemental table 14**: Cecum tissue gene expression data expressed as means +/- SEM and analyzed by Kruskal-Wallis test with FDR corrected *p* values (genes with *p* values < 0.05 in bold).

|  | Untreated – | Untreated – | Untreated – | *B.p.* MP80 – | *B.p.* MP80 – | 2’FL – |
| --- | --- | --- | --- | --- | --- | --- |
|  | *B.p.* MP80 | 2’FL | *B.p.* MP80 | 2’FL | *B.p.* MP80 | *B.p.* MP80 |
|  |  |  | 2'FL |  | 2’FL | 2’FL |
| Cd14 | 0.054 | 0.670 | 0.028 | 0.870 | **0.009** | **0.034** |
| Cyp1a1 | 0.700 | 0.870 | **0.028** | 0.210 | **0.009** | 0.062 |
| Gpx2 | 0.950 | **0.015** | **0.008** | **0.023** | **0.014** | 0.530 |
| Hmox1 | 0.600 | **0.008** | **0.008** | 0.450 | 0.530 | 0.870 |
| Il10 | 0.068 | **0.022** | 0.068 | 0.710 | 0.950 | 0.810 |
| Il10ra | **0.039** | 0.068 | **0.010** | 0.870 | 0.630 | 0.360 |
| Il6 | **0.022** | 0.290 | 0.086 | 0.073 | 0.710 | 0.360 |
| Myd88 | 0.290 | **0.008** | **0.009** | **0.009** | **0.009** | 0.530 |
| Nqo1 | 0.720 | **0.028** | **0.008** | 0.073 | **0.009** | 0.530 |
| Pfkb3 | 0.910 | **0.009** | **0.010** | **0.014** | **0.023** | 0.950 |
| Slc2a1 | 0.350 | **0.008** | **0.008** | **0.009** | **0.009** | 0.870 |

**Supplemental table 15**: Cecum tissue gene expression *p* values calculated by post hoc Wilcoxon Rank Sum test with FDR corrected *p* values. Genes selected based on Kruskal Wallis test with FDR correction and bold values are *p* < 0.05.

|  | Untreated | | |  | *B.p* MP80 | | |  | 2'FL | | |  | *B.p* MP80 + 2'FL | | |
| --- | --- | --- | --- | --- | --- | --- | --- | --- | --- | --- | --- | --- | --- | --- | --- |
| AhR | 1.00 | ± | 0.15 |  | 0.96 | ± | 0.14 |  | 1.24 | ± | 0.40 |  | 0.94 | ± | 0.15 |
| Ccl2 | 1.23 | ± | 0.34 |  | 1.03 | ± | 0.25 |  | 0.93 | ± | 0.18 |  | 1.53 | ± | 0.27 |
| Cd14 | 1.08 | ± | 0.23 |  | 9.26 | ± | 1.46 |  | 1.19 | ± | 0.19 |  | 1.60 | ± | 0.43 |
| Cyp1a1 | 1.40 | ± | 0.44 |  | 0.77 | ± | 0.11 |  | 1.76 | ± | 0.39 |  | 1.54 | ± | 0.44 |
| Gpx2 | 1.02 | ± | 0.10 |  | 1.08 | ± | 0.11 |  | 1.56 | ± | 0.15 |  | 1.85 | ± | 0.25 |
| Hmox1 | 0.86 | ± | 0.12 |  | 0.77 | ± | 0.12 |  | 0.90 | ± | 0.08 |  | 0.77 | ± | 0.09 |
| Hrh2 | 0.84 | ± | 0.33 |  | 1.09 | ± | 0.23 |  | 1.06 | ± | 0.40 |  | 2.75 | ± | 1.44 |
| Il10ra | 0.95 | ± | 0.14 |  | 0.85 | ± | 0.16 |  | 1.08 | ± | 0.24 |  | 0.98 | ± | 0.23 |
| Il1b | 1.17 | ± | 0.33 |  | 0.96 | ± | 0.20 |  | 0.83 | ± | 0.21 |  | 0.78 | ± | 0.24 |
| Il22 | 1.08 | ± | 0.17 |  | 1.27 | ± | 0.48 |  | 1.33 | ± | 0.56 |  | 1.20 | ± | 0.07 |
| Il6 | 1.06 | ± | 0.16 |  | 0.83 | ± | 0.09 |  | 1.28 | ± | 0.14 |  | 1.82 | ± | 0.36 |
| Lcn2 | 3.67 | ± | 1.95 |  | 1.44 | ± | 0.47 |  | 1.46 | ± | 0.98 |  | 10.39 | ± | 6.12 |
| Myd88 | 0.99 | ± | 0.16 |  | 0.76 | ± | 0.08 |  | 0.82 | ± | 0.27 |  | 0.61 | ± | 0.15 |
| Nqo1 | 1.02 | ± | 0.11 |  | 1.44 | ± | 0.10 |  | 1.25 | ± | 0.18 |  | 1.21 | ± | 0.08 |
| Pfkb3 | 0.94 | ± | 0.24 |  | 0.94 | ± | 0.17 |  | 1.71 | ± | 0.51 |  | 0.71 | ± | 0.18 |
| Tgfb | 1.01 | ± | 0.08 |  | 0.90 | ± | 0.11 |  | 1.15 | ± | 0.11 |  | 1.02 | ± | 0.04 |
| Tnfa | 0.99 | ± | 0.31 |  | 0.89 | ± | 0.22 |  | 2.12 | ± | 1.26 |  | 2.26 | ± | 1.01 |

**Supplemental table 16**: Liver tissue gene expression data expressed as means +/- SEM and analyzed by Kruskal-Wallis test with FDR corrected *p* values (genes with *p* values < 0.05 in bold).

|  | Untreated | | | | DSS | | | | DSS +*B.p.* MP80 | | | | DSS +2’FL | | | | DSS +*B.p.* MP80 + 2’FL | | | | |
| --- | --- | --- | --- | --- | --- | --- | --- | --- | --- | --- | --- | --- | --- | --- | --- | --- | --- | --- | --- | --- | --- |
| Starting Weight (g) | | 24.0 | ± | 0.5 | | 25.4 | ± | 1.3 | | 25.0 | ± | 0.9 | | 25.9 | ± | 0.1 | | 25.1 | ± | 0.9 |  |
| Ending Weight (g) | | 24.9 | ± | 0.2 | | 21.9 | ± | 1.4 | | 22.7 | ± | 0.9 | | 24.3 | ± | 0.5 | | 24.5 | ± | 0.3 |  |
| Body Weight Change (%) | | 3.6 | ± | 1.4 ^c^ | | -16.1 | ± | 1.4 ^d^ | | -10.2 | ± | 2.4 ^a^ | | -6.4 | ± | 1.7 ^ab^ | | -2.3 | ± | 2.3 ^b^ |  |
| Spleen Weight (mg/g) | | 3.0 | ± | 0.2 ^b^ | | 5.7 | ± | 0.3 ^a^ | | 6.3 | ± | 0.8 ^a^ | | 5.6 | ± | 0.9 ^a^ | | 3.6 | ± | 0.6 ^b^ |  |
| Liver Weight (mg/g) | | 24.9 | ± | 0.2 ^b^ | | 49.7 | ± | 0.8 ^a^ | | 48.8 | ± | 1.4 ^a^ | | 49.1 | ± | 4.4 ^a^ | | 51.9 | ± | 2.9 ^a^ |  |
| Colon Length (cm) | | 7.3 | ± | 0.3 ^b^ | | 5.5 | ± | 0.3 ^a^ | | 5.3 | ± | 0.3 ^a^ | | 6.2 | ± | 0.4 ^ac^ | | 6.5 | ± | 0.3 ^c^ |  |
| Cecum Weight (w/ content) (mg/g) | | 18.5 | ± | 0.4 ^a^ | | 11.2 | ± | 2.1 ^a^ | | 13.1 | ± | 2.8 ^a^ | | 47.8 | ± | 10.1 ^b^ | | 50.0 | ± | 8.6 ^b^ |  |
| Cecum Weight (w/o content) (mg/g) | | 4.7 | ± | 0.2 ^a^ | | 7.5 | ± | 1.2 ^ab^ | | 6.0 | ± | 1.4 ^ab^ | | 8.7 | ± | 0.4 ^b^ | | 9.6 | ± | 0.6 ^b^ |  |
| Fluid intake before DSS (ml/day/cage) | | 9.3 | ± | 1.3 | | 12.8 | ± | 2.4 | | 12.2 | ± | 1.7 | | 10.2 | ± | 0.8 | | 8.2 | ± | 0.5 |  |
| Fluid intake during DSS (ml/day/cage) | | 10.9 | ± | 2.5 | | 13.9 | ± | 2.6 | | 13.8 | ± | 2.8 | | 9.1 | ± | 1.4 | | 12.4 | ± | 1.8 |  |
| Food intake before DSS (g/day/cage) | | 9.2 | ± | 0.4 | | 8.9 | ± | 1.0 | | 10.3 | ± | 1.1 | | 7.9 | ± | 0.7 | | 7.8 | ± | 1.0 |  |
| Food intake during DSS (g/day/cage) | | 9.6 | ± | 2.0 ^a^ | | 8.6 | ± | 1.5 ^b^ | | 10.4 | ± | 2.1 ^a^ | | 7.8 | ± | 0.6 ^bc^ | | 7.6 | ± | 0.6 ^c^ |  |

**Supplemental table 17**: DSS-colitis experiment mouse physiological data expressed as means +/- SEM and analyzed by one-way ANOVA with FDR corrected *p* values (different letters signify statistical difference between treatments).

|  | Untreated | | |  | DSS | | |  | DSS *+ B.p* MP80 | | |  | DSS + 2'FL | | |  | *DSS + B.p* MP80 + 2'FL | | |
| --- | --- | --- | --- | --- | --- | --- | --- | --- | --- | --- | --- | --- | --- | --- | --- | --- | --- | --- | --- |
| **AhR** | 1.06 | ± | 0.17 |  | 0.25 | ± | 0.05 |  | 0.04 | ± | 0.02 |  | 0.49 | ± | 0.17 |  | 0.51 | ± | 0.09 |
| Bdef2 | 1.12 | ± | 0.21 |  | 2.55 | ± | 0.68 |  | 59.42 | ± | 36.54 |  | 3.61 | ± | 2.87 |  | 1.72 | ± | 0.87 |
| **Ccl2** | 1.07 | ± | 0.17 |  | 10.41 | ± | 3.37 |  | 10.53 | ± | 3.34 |  | 5.82 | ± | 3.15 |  | 1.82 | ± | 0.65 |
| Cd14 | 1.06 | ± | 0.18 |  | 1.53 | ± | 0.21 |  | 1.89 | ± | 0.68 |  | 2.64 | ± | 0.51 |  | 1.37 | ± | 0.32 |
| Foxp3 | 1.03 | ± | 0.12 |  | 7.36 | ± | 2.86 |  | 10.53 | ± | 7.57 |  | 2.19 | ± | 1.64 |  | 0.97 | ± | 0.22 |
| Gpx2 | 1.16 | ± | 0.30 |  | 1.63 | ± | 0.54 |  | 2.49 | ± | 0.85 |  | 1.92 | ± | 0.38 |  | 1.48 | ± | 0.36 |
| Hmox1 | 1.07 | ± | 0.17 |  | 1.86 | ± | 0.31 |  | 2.01 | ± | 0.78 |  | 1.43 | ± | 0.14 |  | 1.88 | ± | 0.57 |
| Hrh2 | 1.05 | ± | 0.16 |  | 9.43 | ± | 3.40 |  | 5.43 | ± | 0.96 |  | 3.68 | ± | 2.95 |  | 0.94 | ± | 0.14 |
| Il10 | 1.15 | ± | 0.27 |  | 1.31 | ± | 0.21 |  | 1.21 | ± | 0.39 |  | 1.11 | ± | 0.37 |  | 1.35 | ± | 0.30 |
| Il10ra | 1.05 | ± | 0.14 |  | 1.32 | ± | 0.53 |  | 9.97 | ± | 5.00 |  | 2.67 | ± | 1.76 |  | 1.56 | ± | 0.50 |
| **Il1b** | 1.07 | ± | 0.19 |  | 19.94 | ± | 6.03 |  | 26.41 | ± | 16.13 |  | 20.87 | ± | 7.59 |  | 5.65 | ± | 2.19 |
| Il22 | 1.13 | ± | 0.26 |  | 27.37 | ± | 7.39 |  | 209.94 | ± | 155.77 |  | 147.61 | ± | 93.73 |  | 94.66 | ± | 65.21 |
| **Il6** | 1.41 | ± | 0.50 |  | 30.95 | ± | 11.93 |  | 75.32 | ± | 30.11 |  | 34.93 | ± | 14.28 |  | 2.11 | ± | 0.82 |
| Lcn2 | 1.02 | ± | 0.08 |  | 5.58 | ± | 1.89 |  | 26.50 | ± | 14.10 |  | 2.74 | ± | 0.48 |  | 1.86 | ± | 0.39 |
| Myd88 | 1.06 | ± | 0.16 |  | 1.07 | ± | 0.24 |  | 1.59 | ± | 0.29 |  | 1.64 | ± | 0.84 |  | 1.05 | ± | 0.20 |
| Nqo1 | 1.08 | ± | 0.18 |  | 0.40 | ± | 0.29 |  | 0.68 | ± | 0.23 |  | 0.51 | ± | 0.08 |  | 0.75 | ± | 0.22 |
| **Ocln** | 1.05 | ± | 0.15 |  | 0.33 | ± | 0.06 |  | 0.31 | ± | 0.06 |  | 0.45 | ± | 0.11 |  | 0.91 | ± | 0.20 |
| Pfkb3 | 1.01 | ± | 0.07 |  | 0.78 | ± | 0.22 |  | 0.89 | ± | 0.76 |  | 0.63 | ± | 0.14 |  | 1.41 | ± | 0.88 |
| Slc2a1 | 1.25 | ± | 0.18 |  | 0.38 | ± | 0.12 |  | 0.68 | ± | 0.58 |  | 0.30 | ± | 0.08 |  | 0.31 | ± | 0.06 |
| Slc6a4 | 1.08 | ± | 0.19 |  | 0.39 | ± | 0.18 |  | 0.68 | ± | 0.23 |  | 2.15 | ± | 0.99 |  | 3.10 | ± | 1.33 |
| **Tgfb** | 1.04 | ± | 0.13 |  | 0.38 | ± | 0.08 |  | 0.11 | ± | 0.05 |  | 0.57 | ± | 0.09 |  | 0.73 | ± | 0.19 |
| Tnfa | 1.21 | ± | 0.35 |  | 2.40 | ± | 0.70 |  | 6.53 | ± | 2.21 |  | 6.87 | ± | 2.11 |  | 3.31 | ± | 1.07 |
| Tph1 | 1.08 | ± | 0.18 |  | 0.49 | ± | 0.07 |  | 0.51 | ± | 0.22 |  | 0.52 | ± | 0.09 |  | 0.92 | ± | 0.17 |

**Supplemental table 18**: Colon tissue gene expression data from chemically-induced colitis experiments expressed as means +/- SEM and are analyzed by Kruskal-Wallis test with FDR corrected *p* values (genes with *p* values < 0.05 in bold).

|  |  |  |  |  |  |  | |  |  | DSS | DSS |
| --- | --- | --- | --- | --- | --- | --- | --- | --- | --- | --- | --- |
|  |  |  |  | Untreated |  |  | DSS – | | DSS | *B.p.* MP80 – | 2FL – |
|  |  | Untreated | Untreated | – DSS | DSS – | DSS – | DSS | | *B.p.* MP80 – | DSS | DSS |
|  | Untreated | – DSS | – DSS | *B.p.* MP80 | DSS | DSS | *B.p.* MP80 | | DSS | *B.p.* MP80 | *B.p.* MP80 |
|  | – DSS | *B.p.* MP80 | 2'FL | 2FL | MP80 | 2FL | 2FL | | 2FL | 2FL | 2FL |
| AhR | **0.024** | **0.024** | 0.110 | **0.024** | **0.032** | 0.490 | **0.036** | | **0.024** | **0.024** | 0.490 |
| Ccl2 | **0.024** | **0.024** | **0.024** | 0.650 | 0.950 | 0.200 | **0.024** | | 0.650 | **0.050** | 0.110 |
| Il1b | **0.024** | **0.024** | **0.050** | **0.024** | 0.630 | 0.950 | 0.073 | | 0.830 | 0.190 | 0.490 |
| Il6 | **0.024** | **0.024** | **0.024** | 0.520 | 0.320 | 1.000 | **0.024** | | 0.260 | **0.024** | **0.040** |
| Ocln | **0.024** | **0.024** | **0.024** | 0.750 | 0.750 | 0.580 | **0.050** | | 0.490 | 0.073 | 0.150 |
| Tgfb | **0.024** | **0.024** | 0.057 | 0.450 | **0.048** | 0.450 | 0.190 | | **0.031** | **0.047** | 0.630 |

**Supplemental table 19**: Colon tissue gene expression data from chemically-induced colitis experiments *p* values calculated by post hoc Wilcoxon Rank Sum test with FDR corrected *p* values. Genes selected based on Kruskal Wallis test with FDR correction and bold values are *p* < 0.05.

|  | Untreated | | |  | DSS | | |  | DSS *+ B.p* MP80 | | |  | DSS + 2'FL | | |  | *DSS + B.p* MP80 + 2'FL | | |
| --- | --- | --- | --- | --- | --- | --- | --- | --- | --- | --- | --- | --- | --- | --- | --- | --- | --- | --- | --- |
| **AhR** | 1.02 | ± | 0.09 |  | 0.51 | ± | 0.09 |  | 0.47 | ± | 0.11 |  | 0.94 | ± | 0.14 |  | 0.45 | ± | 0.11 |
| Ccl2 | 1.07 | ± | 0.18 |  | 1.14 | ± | 0.24 |  | 0.86 | ± | 0.17 |  | 0.94 | ± | 0.18 |  | 0.78 | ± | 0.17 |
| Cd14 | 1.05 | ± | 0.15 |  | 0.60 | ± | 0.13 |  | 0.76 | ± | 0.10 |  | 0.93 | ± | 0.10 |  | 0.65 | ± | 0.11 |
| Foxp3 | 1.04 | ± | 0.13 |  | 0.66 | ± | 0.10 |  | 0.62 | ± | 0.09 |  | 1.08 | ± | 0.19 |  | 0.72 | ± | 0.16 |
| Gpx2 | 1.45 | ± | 0.59 |  | 0.52 | ± | 0.21 |  | 0.79 | ± | 0.20 |  | 0.63 | ± | 0.24 |  | 0.62 | ± | 0.27 |
| Hmox1 | 0.89 | ± | 0.19 |  | 0.23 | ± | 0.08 |  | 0.49 | ± | 0.09 |  | 0.41 | ± | 0.07 |  | 0.45 | ± | 0.09 |
| Hrh2 | 1.05 | ± | 0.13 |  | 0.70 | ± | 0.08 |  | 0.76 | ± | 0.17 |  | 1.28 | ± | 0.28 |  | 0.69 | ± | 0.18 |
| Il10 | 1.05 | ± | 0.14 |  | 0.30 | ± | 0.11 |  | 0.56 | ± | 0.15 |  | 0.53 | ± | 0.11 |  | 0.36 | ± | 0.09 |
| Il10ra | 1.01 | ± | 0.07 |  | 0.39 | ± | 0.07 |  | 0.82 | ± | 0.26 |  | 0.91 | ± | 0.20 |  | 0.72 | ± | 0.19 |
| IL1b | 1.12 | ± | 0.21 |  | 0.26 | ± | 0.11 |  | 0.53 | ± | 0.11 |  | 0.71 | ± | 0.14 |  | 0.57 | ± | 0.13 |
| Il22 | 1.02 | ± | 0.12 |  | 0.82 | ± | 0.35 |  | 33.64 | ± | 30.74 |  | 2.81 | ± | 1.01 |  | 5.60 | ± | 4.52 |
| Il6 | 1.03 | ± | 0.11 |  | 1.61 | ± | 0.46 |  | 1.69 | ± | 0.28 |  | 1.40 | ± | 0.25 |  | 2.03 | ± | 0.46 |
| Lcn2 | 1.20 | ± | 0.37 |  | 1.36 | ± | 0.39 |  | 1.65 | ± | 0.44 |  | 1.82 | ± | 0.50 |  | 0.84 | ± | 0.25 |
| Myd88 | 1.29 | ± | 0.37 |  | 0.33 | ± | 0.15 |  | 0.68 | ± | 0.11 |  | 0.52 | ± | 0.18 |  | 0.59 | ± | 0.17 |
| **Nqo1** | 1.01 | ± | 0.07 |  | 0.24 | ± | 0.10 |  | 0.33 | ± | 0.09 |  | 1.19 | ± | 0.62 |  | 1.53 | ± | 0.69 |
| Ocln | 1.01 | ± | 0.16 |  | 0.35 | ± | 0.13 |  | 0.52 | ± | 0.08 |  | 0.72 | ± | 0.18 |  | 0.63 | ± | 0.16 |
| **Pfkb3** | 1.16 | ± | 0.30 |  | 1.73 | ± | 0.28 |  | 2.25 | ± | 0.87 |  | 0.54 | ± | 0.18 |  | 0.35 | ± | 0.08 |
| Slc2a1 | 1.01 | ± | 0.08 |  | 0.60 | ± | 0.13 |  | 0.57 | ± | 0.09 |  | 0.74 | ± | 0.14 |  | 0.45 | ± | 0.08 |
| Slc6a4 | 0.90 | ± | 0.16 |  | 0.32 | ± | 0.08 |  | 0.51 | ± | 0.08 |  | 0.59 | ± | 0.08 |  | 0.51 | ± | 0.12 |
| **Tgfb** | 1.13 | ± | 0.15 |  | 0.44 | ± | 0.07 |  | 0.51 | ± | 0.08 |  | 0.77 | ± | 0.11 |  | 0.51 | ± | 0.08 |
| Tnfa | 1.14 | ± | 0.35 |  | 0.54 | ± | 0.24 |  | 3.02 | ± | 1.88 |  | 0.24 | ± | 0.06 |  | 0.37 | ± | 0.10 |
| Tph1 | 0.89 | ± | 0.14 |  | 0.24 | ± | 0.07 |  | 0.42 | ± | 0.07 |  | 0.55 | ± | 0.09 |  | 0.48 | ± | 0.10 |

**Supplemental table 20**: Jejunum tissue gene expression data from chemically-induced colitis experiments expressed as means +/- SEM and are analyzed by Kruskal-Wallis test with FDR corrected *p* values (genes with *p* values < 0.05 in bold).

|  |  |  |  |  |  |  |  |  | | DSS | | DSS |
| --- | --- | --- | --- | --- | --- | --- | --- | --- | --- | --- | --- | --- |
|  |  |  |  | Untreated |  |  | DSS – | DSS | | *B.p.* MP80 – | | 2FL – |
|  |  | Untreated | Untreated | – DSS | DSS – | DSS – | DSS | *B.p.* MP80 – | | DSS | | DSS |
|  | Untreated | – DSS | – DSS | *B.p.* MP80 | DSS | DSS | *B.p.* MP80 | DSS | | *B.p.* MP80 | | *B.p.* MP80 |
|  | – DSS | *B.p.* MP80 | 2'FL | 2FL | MP80 | 2FL | 2FL | 2FL | | 2FL | | 2FL |
| AhR | **0.019** | **0.019** | 1.000 | **0.027** | 0.910 | 0.065 | 0.820 | | **0.065** | 0.990 | 0.160 | |
| Nqo1 | **0.019** | **0.019** | 0.610 | 0.910 | 0.640 | 0.130 | **0.027** | | 0.480 | **0.027** | 0.490 | |
| Pfkb3 | 0.160 | 0.640 | 0.097 | **0.017** | 0.910 | **0.027** | **0.017** | | 0.130 | **0.043** | 0.710 | |
| Tgfb | **0.015** | **0.017** | 0.150 | **0.017** | 0.640 | 0.100 | 0.640 | | 0.290 | 1.000 | 0.190 | |

**Supplemental table 21**: Jejunum tissue gene expression data from chemically-induced colitis experiments *p* values calculated by post hoc Wilcoxon Rank Sum test with FDR corrected *p* values. Genes selected based on Kruskal Wallis test with FDR correction and bold values are *p* < 0.05.

|  | Untreated | | |  | DSS | | |  | DSS *+ B.p* MP80 | | |  | DSS + 2'FL | | |  | *DSS + B.p* MP80 + 2'FL | | |
| --- | --- | --- | --- | --- | --- | --- | --- | --- | --- | --- | --- | --- | --- | --- | --- | --- | --- | --- | --- |
| **AhR** | 1.01 | ± | 0.06 |  | 0.3383 | ± | 0.046 |  | 0.2792 | ± | 0.0597 |  | 0.443 | ± | 0.097 |  | 0.541 | ± | 0.08 |
| Ccl2 | 1.09 | ± | 0.2 |  | 1.8844 | ± | 0.353 |  | 3.8259 | ± | 1.3452 |  | 2.0459 | ± | 0.506 |  | 1.448 | ± | 0.46 |
| **Cd14** | 1.01 | ± | 0.07 |  | 2.0541 | ± | 0.673 |  | 3.8954 | ± | 0.952 |  | 1.2344 | ± | 0.29 |  | 0.921 | ± | 0.1 |
| Gpx2 | 1.11 | ± | 0.18 |  | 0.9351 | ± | 0.341 |  | 0.3561 | ± | 0.0748 |  | 0.5002 | ± | 0.077 |  | 0.843 | ± | 0.24 |
| Hmox1 | 1.27 | ± | 0.39 |  | 1.0577 | ± | 0.437 |  | 0.835 | ± | 0.1909 |  | 0.7494 | ± | 0.089 |  | 0.811 | ± | 0.17 |
| Hrh2 | 1.02 | ± | 0.09 |  | 0.8353 | ± | 0.392 |  | 0.8646 | ± | 0.1706 |  | 0.7094 | ± | 0.164 |  | 0.629 | ± | 0.17 |
| **Il10ra** | 1.01 | ± | 0.06 |  | 0.2225 | ± | 0.034 |  | 0.2984 | ± | 0.0488 |  | 0.4066 | ± | 0.091 |  | 0.401 | ± | 0.03 |
| Il1b | 1.29 | ± | 0.41 |  | 1.5637 | ± | 0.818 |  | 0.9813 | ± | 0.3397 |  | 1.1802 | ± | 0.082 |  | 1.471 | ± | 0.28 |
| **Il6** | 1.04 | ± | 0.14 |  | 2.6899 | ± | 0.411 |  | 2.0999 | ± | 0.3979 |  | 1.0022 | ± | 0.299 |  | 1.659 | ± | 0.41 |
| **Lcn2** | 1.47 | ± | 0.5 |  | 234.73 | ± | 93.32 |  | 462.1 | ± | 89.649 |  | 189.49 | ± | 78.75 |  | 11.4 | ± | 3.45 |
| Myd88 | 1.31 | ± | 0.38 |  | 0.5961 | ± | 0.204 |  | 0.7929 | ± | 0.3084 |  | 0.5518 | ± | 0.059 |  | 0.755 | ± | 0.22 |
| Nqo1 | 0.86 | ± | 0.14 |  | 0.2545 | ± | 0.12 |  | 0.2283 | ± | 0.0613 |  | 0.3356 | ± | 0.04 |  | 0.493 | ± | 0.11 |
| Pfkb3 | 1.02 | ± | 0.09 |  | 1.1991 | ± | 0.751 |  | 1.1343 | ± | 0.4344 |  | 0.4304 | ± | 0.091 |  | 0.361 | ± | 0.13 |
| **Slc2a1** | 1.06 | ± | 0.17 |  | 0.1919 | ± | 0.083 |  | 0.1746 | ± | 0.0427 |  | 0.3673 | ± | 0.121 |  | 0.509 | ± | 0.09 |
| **Tgfb** | 1.01 | ± | 0.07 |  | 0.353 | ± | 0.071 |  | 0.3818 | ± | 0.0593 |  | 0.5457 | ± | 0.067 |  | 0.669 | ± | 0.06 |
| Tnfa | 1.08 | ± | 0.35 |  | 0.4499 | ± | 0.2 |  | 0.7323 | ± | 0.3258 |  | 0.4991 | ± | 0.211 |  | 0.777 | ± | 0.22 |

**Supplemental table 22**: Liver tissue gene expression data from chemically-induced colitis experiments expressed as means +/- SEM and are analyzed by Kruskal-Wallis test with FDR corrected *p* values (genes with *p* values < 0.05 in bold).

|  |  |  |  |  |  |  |  |  | DSS | DSS |
| --- | --- | --- | --- | --- | --- | --- | --- | --- | --- | --- |
|  |  |  |  | Untreated |  |  | DSS – | DSS | *B.p.* MP80 – | 2FL – |
|  |  | Untreated | Untreated | – DSS | DSS – | DSS – | DSS | *B.p.* MP80 – | DSS | DSS |
|  | Untreated | – DSS | – DSS | *B.p.* MP80 | DSS | DSS | *B.p.* MP80 | DSS | *B.p.* MP80 | *B.p.* MP80 |
|  | – DSS | *B.p.* MP80 | 2'FL | 2FL | MP80 | 2FL | 2FL | 2FL | 2FL | 2FL |
| AhR | **0.009** | **0.009** | **0.009** | **0.009** | 0.660 | 0.660 | 0.160 | 0.410 | 0.053 | 0.480 |
| Cd14 | 0.340 | **0.009** | 0.940 | 0.480 | 0.210 | 0.660 | 0.210 | **0.013** | **0.009** | 0.870 |
| Il10ra | **0.009** | **0.009** | **0.009** | **0.009** | 0.480 | 0.340 | **0.009** | 0.410 | 0.160 | 0.940 |
| Il6 | **0.013** | **0.020** | 0.750 | 0.340 | 0.410 | **0.038** | 0.350 | 0.100 | 0.720 | 0.340 |
| Lcn2 | **0.009** | **0.013** | **0.009** | **0.020** | 0.210 | 0.940 | **0.013** | 0.061 | **0.013** | 0.053 |
| Slc2a1 | **0.009** | **0.013** | **0.013** | **0.020** | 0.720 | 0.160 | 0.120 | 0.640 | **0.020** | 0.480 |
| Tgfb | **0.009** | **0.009** | **0.009** | **0.020** | 0.940 | 0.280 | **0.020** | 0.160 | **0.020** | 0.480 |

**Supplemental table 23**: Liver tissue gene expression data from chemically-induced colitis experiments *p* values calculated by post hoc Wilcoxon Rank Sum test with FDR corrected *p* values. Genes selected based on Kruskal Wallis test with FDR correction and bold values are *p* < 0.05.

|  | Spearman's Correlation | | *p* value | |
| --- | --- | --- | --- | --- |
| Slc6a4 | 0.104 | 0.748 | |  |
| Tph1 | 0.799 | **0.006** | |  |
| AhR | 0.786 | **0.004** | |  |
| Nqo1 | 0.158 | 0.643 | |  |
| Hmox1 | 0.104 | 0.761 | |  |
| Ocln | 0.441 | 0.151 | |  |
| Il10ra | -0.104 | 0.748 | |  |
| Il1b | -0.095 | 0.782 | |  |
| Tnf | -0.295 | 0.351 | |  |
| Il6 | -0.723 | **0.018** | |  |
| Ccl2 | -0.478 | 0.116 | |  |
| Cd14 | -0.250 | 0.434 | |  |
| Hrh2 | -0.549 | 0.080 | |  |
| Lcn2 | -0.300 | 0.344 | |  |
| Tgfb | 0.800 | **0.005** | |  |
| Nfkb | 0.624 | **0.030** | |  |
| Colon Length | 0.815 | **0.001** | |  |
| LBP in Serum | -0.724 | **0.008** | |  |
| Spleen Weight | -0.528 | 0.077 | |  |

**Supplemental table 24**: Spearman’s correlation between colon inflammatory markers and *B.p.* MP80 abundance as measured by qPCR with a fucosidase primer for treatment groups DSS + *B.p.* MP80 + 2’FL and DSS + *B.p.* MP80 (*n* = 6 per treatment group).
